# Finding Web-Based Anxiety Interventions on the World Wide Web: A Scoping Review

**DOI:** 10.2196/mental.5349

**Published:** 2016-06-01

**Authors:** Miriam Thiel Ashford, Ellinor K Olander, Susan Ayers

**Affiliations:** ^1^ Centre for Maternal and Child Health Research School of Health Sciences City University London London United Kingdom

**Keywords:** Anxiety, mental health, web-based interventions, internet, technology, consumer, access to health care

## Abstract

**Background:**

One relatively new and increasingly popular approach of increasing access to treatment is Web-based intervention programs. The advantage of Web-based approaches is the accessibility, affordability, and anonymity of potentially evidence-based treatment. Despite much research evidence on the effectiveness of Web-based interventions for anxiety found in the literature, little is known about what is publically available for potential consumers on the Web.

**Objective:**

Our aim was to explore what a consumer searching the Web for Web-based intervention options for anxiety-related issues might find. The objectives were to identify currently publically available Web-based intervention programs for anxiety and to synthesize and review these in terms of (1) website characteristics such as credibility and accessibility; (2) intervention program characteristics such as intervention focus, design, and presentation modes; (3) therapeutic elements employed; and (4) published evidence of efficacy.

**Methods:**

Web keyword searches were carried out on three major search engines (Google, Bing, and Yahoo—UK platforms). For each search, the first 25 hyperlinks were screened for eligible programs. Included were programs that were designed for anxiety symptoms, currently publically accessible on the Web, had an online component, a structured treatment plan, and were available in English. Data were extracted for website characteristics, program characteristics, therapeutic characteristics, as well as empirical evidence. Programs were also evaluated using a 16-point rating tool.

**Results:**

The search resulted in 34 programs that were eligible for review. A wide variety of programs for anxiety, including specific anxiety disorders, and anxiety in combination with stress, depression, or anger were identified and based predominantly on cognitive behavioral therapy techniques. The majority of websites were rated as credible, secure, and free of advertisement. The majority required users to register and/or to pay a program access fee. Half of the programs offered some form of paid therapist or professional support. Programs varied in treatment length and number of modules and employed a variety of presentation modes. Relatively few programs had published research evidence of the intervention’s efficacy.

**Conclusions:**

This review represents a snapshot of available Web-based intervention programs for anxiety that could be found by consumers in March 2015. The consumer is confronted with a diversity of programs, which makes it difficult to identify an appropriate program. Limited reports and existence of empirical evidence for efficacy make it even more challenging to identify credible and reliable programs. This highlights the need for consistent guidelines and standards on developing, providing, and evaluating Web-based interventions and platforms with reliable up-to-date information for professionals and consumers about the characteristics, quality, and accessibility of Web-based interventions.

## Introduction

The National Comorbidity Survey Replication showed that 28.8% of people in the United States suffer from an anxiety disorder in their lifetime [1]. Reviews suggest that anxiety disorders are the most frequently occurring class of mental health disorders [2-4] and are considered chronic and disabling conditions worldwide [5]. Despite effective treatments being available, anxiety disorders are still widely underdiagnosed and undertreated [4,6]. The adverse effects of anxiety disorders on psychological and somatic health, as well as high economic costs [2,6-8] mean that treatment is a significant public health issue.

Lack of help-seeking behavior and perceived barriers to accessing treatment contribute to underdiagnosis and undertreatment. Generally, individuals with anxiety display a tendency not to seek help for their disorder [9,10]. Identified treatment barriers include lack of awareness of the presence of a disorder and available services, financial burden, and the stigma associated with disclosing mental health disorders [10,11].

Research has shown that many individuals use the Internet to find information or help for health-related topics [12], especially for topics that they experience as difficult to talk about [13,14]. A survey demonstrated that 18% of all surveyed Internet users had searched the Internet for mental health-related information, with higher prevalence for those who had a history of mental health issues and those who at the time stated that they were experiencing psychological distress [15]. Similarly, nationally representative surveys from the Pew Internet and American Life Project found that 26-39% of individuals who sought Web-based health information looked at mental health information [14,16]. When searching the Internet for mental health information, individuals may come across Web-based interventions.

A Web-based intervention has been defined as “a primarily self-guided intervention program that is executed by means of a prescriptive online program operated through a website.” (p. 5) [17]. Advantages of Web-based approaches include accessibility, affordability, and anonymity of mental health interventions [18,19]. Web-based interventions can be accessed anytime and anywhere from devices such as computers, laptops, tablets, and mobile phones and large audiences and rural areas can be reached in a cost-effective manner [20-22]. Web-based interventions also offer anonymity and privacy, which may attract individuals who experience difficulties with disclosing mental health disorders [23-24].

The efficacy of Web-based mental health intervention programs is well established. Meta-analyses of Web-based mental health interventions have shown that those interventions were as effective as face-to-face treatments and superior to control groups with substantial effect sizes [25,26]. With regard to anxiety disorders specifically, a meta-analysis concluded that computerized- and Internet-based cognitive behavioral therapy (CBT) for anxiety disorders had improved outcomes compared to waitlist and placebo assignments and these effects were equal to face-to-face treatment [27,28]. Another meta-analytic review concluded that computer-aided psychotherapy was as effective as face-to-face therapy and that the effects did not differ across various anxiety disorders and types of delivery [29]. Similarly, a recent review reported moderate to large effect sizes for Internet-based CBT for a range of anxiety disorders ranging from 0.30 to 2.53 [30].

Despite an extensive body of literature evaluating the effectiveness of developed Web-based interventions, little research has examined the range and characteristics of publically available Web-based intervention programs for individuals with mental health issues. Research has started to identify, describe, and evaluate the range and characteristics of mental health mobile phone apps [31,32] and e-therapy or e-counselling services [33,34]. However, e-therapy is different from Web-based programs, as in e-therapy mental health professionals use text- or video-based formats (eg, email, chat, Skype) for delivering therapy. There is also a clinical online directory of Web-based mental health programs called Beacon available, which lists among others, intervention program websites for phobias, generalized anxiety disorder, social anxiety disorder, and panic disorder [35]. However, this website is not updated very often and is clinically directed rather than a systematic review of programs that are publically available. A few publically available Web-based programs were briefly discussed in a review; however, this was restricted to four programs available in Australia (FearFighter, Beating the Blues, Online Anxiety, CRUfAD) and included only a short overall description of the main program characteristics [36]. In addition, the programs were not identified by a systematic Web search. Recently, a scoping review has identified and evaluated currently available interactive Web-based interventions for depression [37]. However, to our knowledge no study has conducted a similar review for Web-based intervention programs for anxiety.

In summary, despite the clear advantages of Web-based anxiety interventions, there is only limited systematically identified and up-to-date information available on the characteristics of publically available Web-based interventions for anxiety and the quality of these services is currently unknown. This information would be helpful and important for consumers and practitioners interested in Web-based interventions for anxiety, as well as researchers developing and evaluating those interventions. Therefore, this study conducted a replicable Web search to identify freely available Web-based anxiety intervention programs and review these in terms of (1) website characteristics such as origin, accessibility, and credibility; (2) Web-based program characteristics, such as intervention focus, design, delivery, and features; (3) intervention characteristics such as the overall therapeutic approach and intervention features; as well as (4) published evidence of efficacy.

## Methods

### Search Strategy

Using the 3 most popular Web search engines, Google, Bing, and Yahoo [38,39], a keyword search for Web-based intervention programs for anxiety was performed in March 2015. UK versions of the search engines were used (.co.uk). Before starting the search, existing search history and cookies were deleted and future tracking and cookies were disabled in the browser. A list of the 9 search term combinations used can be found in Textbox 1. Primarily simple and lay keywords were used to simulate a Web search that was relatively likely to be conducted by an individual searching for Web-based programs. It has been shown that most individuals rarely consider more than the first 20 links generated by a search engine [40]. As featured links placed at the top and bottom were also considered, we chose to assess the first 25 links. This resulted in 675 hyperlinks being screened (3 search engines × 9 search terms × 25 hyperlinks).

**Search terms used in Google, Bing, and Yahoo.**

1. Internet therapy anxiety
2. Internet treatment anxiety
3. Internet cognitive behavioural therapy anxiety
4. Online therapy anxiety
5. Online treatment anxiety
6. Online cognitive behavioural therapy anxiety
7. Web therapy anxiety
8. Web treatment anxiety
9. Web cognitive behavioural therapy anxiety

### Program Identification

All 675 hyperlinks were screened for eligible Web-based programs for anxiety by the first author. The screening process consisted of two stages. The first stage involved screening all 675 hyperlinked websites to eliminate clearly irrelevant websites. All hyperlinks were screened and organized into 1 of the following 3 categories: websites with Web-based programs, websites linking to websites with Web-based programs, and websites with irrelevant content. Irrelevant content included, among others, e-counselling websites, mental health information websites, support groups/forums, online mental health screening/assessment, therapist or mental health clinic websites, scholarly articles, blogs, Facebook pages, Wikipedia, videos, and broken links.

For all websites categorized as “websites of Web-based programs” and “websites linking to websites of Web-based programs,” duplicates were removed. All remaining websites entered the second stage of screening and were screened according to the following criteria by the first author: (1) designed for anxiety symptoms (although they did not need to be focused on anxiety only), (2) currently publically accessible on the Internet (via registration, application, General Practitioner (GP) referral), (3) online component, (4) structured treatment plan (information only), and (5) available in English. Programs were excluded if they were (1) not publically accessible; (2) Web-based counseling only (Skype, email, or instant message contact with a counselor only, with no structured program associated); (3) purely informational (psychoeducation only); or (4) exclusively part of a research study.

### Data Extraction

A data extraction form was created containing 4 main categories and 9 subcategories. Table 1 provides an overview of the items in each main category and subcategories. The 4 main categories are based on the 4 specified study interests (website characteristics, program characteristics, intervention characteristics, and empirical evidence). The 9 subcategories of the 4 main categories were established by incorporating the 12 key facets of a framework designed for evaluating and reporting Internet intervention studies [41]. The extraction was undertaken by the first author in March 2015. Screenshots of all programs and websites were taken in case the program changed during the rating period. To ensure that the programs could be evaluated thoroughly, all program authors were contacted to request free access.

**Table 1:** Data extraction categories and subcategories.

Main Category	Subcategory	Item
Website characteristics	Origin	Country of origin
	Accessibility	Registration (yes/no—if yes, how?)
		Log-in available on website (yes/no)
		Access fee (yes/no—if yes, how much? Free trial available? Refund period? Length of subscription)
		Mobile phone rendering (yes/no)
	Credibility	Advertisements (yes/no—if yes, relevant vs irrelevant)
		Presented contact details (yes/no)
		Specified authorship (yes/no)
		Terms of use specified (yes/no)
		Privacy notice specified (yes/no)
Program characteristics	Intervention focus	Target anxiety issue
		Target audience
	Intervention design	Therapist support (yes/no—if yes, specify)
		Suggested or set treatment length
		Number of modules
	Intervention delivery	Presentation format
Intervention characteristics	Therapeutic approach	CBT^a^; others (specify)
		Other therapeutic elements
	Intervention features	Worksheets (yes/no—if yes, specify format)
		Mood or symptom monitoring (yes/no)
		Diary (yes/no)
		Forum (yes/no)
		Other features (yes/no—if yes, specify)
Empirical evidence	Empirical evidence for program efficacy	Scrutinized program website for relevant information, contacted the author, and checked the Beacon^b^directory


^a^CBT: cognitive behavioral therapy

^b^Beacon: Australian clinical Web-based platform that describes different Web-based self-help treatment programs [49].

### Program Evaluation

Several validated and widely accepted scales are available to evaluate the methodology of published studies. However, there are currently no validated criteria available for evaluating actual Web-based interventions as found on the web. Renton et al [31] created a program scoring system to evaluate Web-based depression interventions. With permission from the authors, the scale was adapted to fit the 4 specified study interests. The adapted version consists of 16 yes or no close-ended questions that are outlined in Table 2. Consistent with the scoring system used by Renton et al [31], a score of 1 was awarded if the answer was yes, and a score of 0 if the answer was no or the question could not be evaluated. Scores were converted into percentages, with higher scores indicating a larger number of met criteria on the scale.

**Table 2:** Program evaluation criteria.

Main Category	Question
Website characteristics	1. Was country of origin stated?
	2. Was a unique user name or password provided to users?
	3. Were the names and credentials of authors present?
	4. Were contact details provided?
	5. Were the Terms of Use specified?
	6. Was a Privacy Notice specified?
	7. Was evidence for the program provided to the user (ie, attrition data/success rate/completion rate/# of users in the program/testimonials)?
Program characteristics	8. Were the primary focus/goals/objectives of the intervention stated?
	9. Was the patient group or target mental health issue specified?
	10. Was the number of modules or time to complete each module stated?
	11. Was the intervention tailored to the user or was it generic for all users?
	12. Did the program offer multimedia content delivery (ie, a combination of text, video, graphics, and audio formats)?
	13. Was the program easy to navigate?
Intervention characteristics	14. Was the model of change (ie, type of therapy utilized) defined/stated?
	15. Was information on what is covered in the intervention modules provided (ie, names of modules or a short description)?
Empirical evidence	16. Has the program been empirically validated?

## Results

### Program Selection

**Figure 1:**
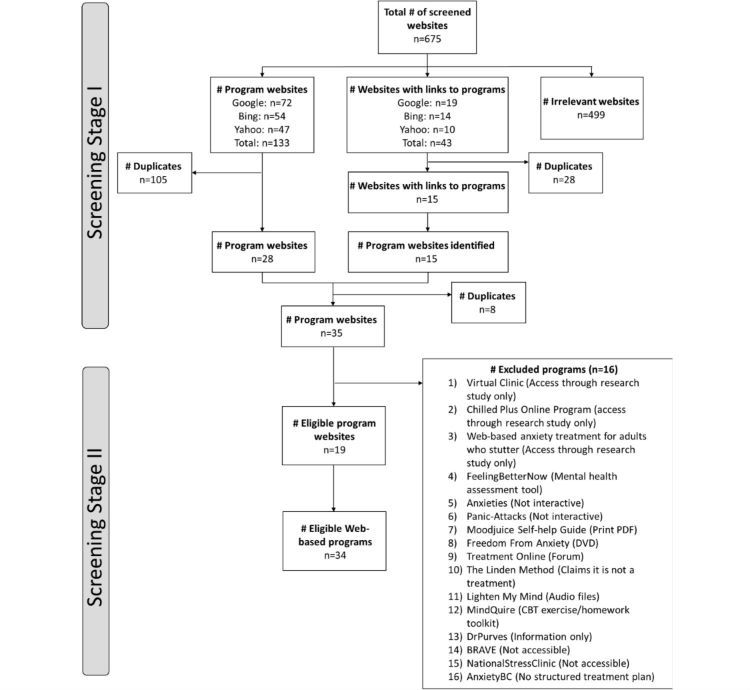
Flow diagram of program selection.

A search log outlines the number of hits per search, as well as the number of included and excluded hyperlinks (see Multimedia Appendix 1). Most program websites were identified when the search terms “online treatment anxiety” (Program websites: n=14, websites with links to program websites: n=2) and “online cognitive behavioural therapy anxiety” (program websites: n=12, websites with links to program websites: n=4) were entered into Google. “Web therapy anxiety” entered in Yahoo did not identify any program websites. All 675 links were assessed for inclusion. Figure 1 displays the flowchart for the two-stage selection process of included programs and reasons for exclusion. In total, 176 of the 675 (25.3%) assessed hyperlinks led either directly to program websites (133/675, 19.7%) or to websites containing links to program websites (43/675, 6.4%). The first stage of the screening identified 35 potentially eligible program websites that were subsequently assessed for inclusion. Of those, 19 websites and 34 programs met the inclusion criteria. For 5 programs, the authors did not grant access and some aspects of those programs could therefore not be evaluated (Beating the Blues, Changing States, eCentreClinic, FearFighter, Social Anxiety Institute).

### Website Characteristics

#### Country of Origin

Programs identified in this review originated from 4 different countries. The majority of programs were developed in Australia (16/34, 47%), followed by the UK (9/34, 28%) and the USA (8/34, 24%), and Sweden with one program.

#### Accessibility

**Figure 2:**
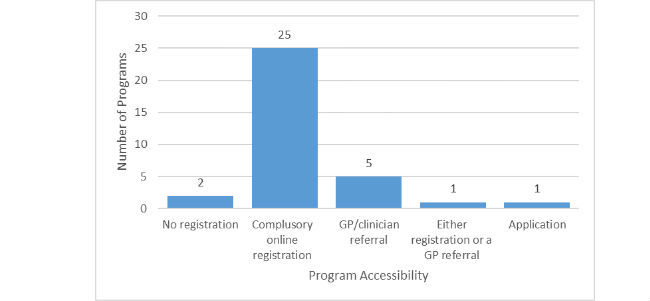
Access to evaluated programs.

Out of the 34 programs evaluated, 25 (74%) had a compulsory online registration process to access the program, 5 (15%) required GP/clinician referral, 1 (3%) was accessible through either registration or GP referral, 1 (3%) through application, and 2 (6%) did not require registration to access the program (see Figure 2). Excluding the 2 programs that did not require registration, 29 (85%) had a log-in feature on their website. For 3 programs (9%), no log-in feature was found and it was unclear how users would log in after buying the program. For programs requiring registration, consumers had to enter personal information to set up a profile. Registration allowed tracking and saving of entered information. This was not possible for programs that required no registration.

Over half of programs (24/34, 71%) required an access fee, while 3 were free if signing up for a research trial. Costs varied from £14.99 (Changing States) for 1 module to £197 (FearFighter) for 9 modules and therapist support. Most programs that required a fee had either a free trial period, or a 100% refund period and were either weekly/monthly subscriptions or only valid for 1 to 6 months. Out of the 34 programs, 9 (28%) were accessible in a mobile phone version, while 3 (9%) could not be evaluated in this respect, as the authors did not grant access to the program.

#### Credibility

**Figure 3:**
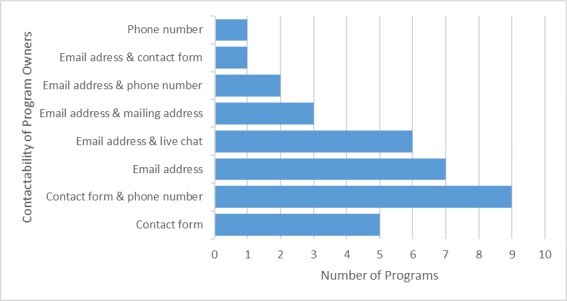
Methods of contacting the program owner.

All programs specified authorship and all programs presented contact details either via a contact form and phone number (9/34, 28%), email address (7/34, 21%), email address and live chat (6/34, 18%), contact form (5/34, 15%), email address and mailing address (3/34,9%), email address and phone number (2/34, 6%), phone number (1/34, 3%), or email address and contact form (1/34, 3%) (see Figure 3). Thirty out of 34 (88%) programs specified their terms of use and 32 (94%) had a privacy notice. All programs with a privacy notice also included information about browser cookies, data collection, and data management. Thirty-one programs (91%) displayed no advertisements. One program’s advertisement was deemed relevant (mental health self-help books) and the other two were deemed irrelevant (BBC news link and Google Ads).

### Intervention Program Characteristics

An overview of intervention program characteristics for each program can be found in Table 3 and screenshots of all programs can be found in Multimedia Appendix 2.

**Table 3:** Intervention program characteristics of included Web-based intervention programs for anxiety.

Program (Ref#)	Target Anxiety Issue & Population	Therapist-Assisted	Structure & Length	Presentation Format	Therapeutic Approach	Intervention Features
AI-Therapy (#1)	Social anxiety	No	7 modules (1-2 modules per week)	- Text chapters with figures - Audio features with every chapter - Video features	CBT^a^	- Online worksheets - Online questionnaires - Symptom tracking - Email reminders - Knowledge quizzes - Personalized eBook
Beating the Blues (#2)	Anxiety & depression	No	8 sessions (over 8 weeks)	- Image slides with audio & video - Interactive slides	CBT^a^	- Worksheet printouts - Email reminders
Blues Begone (#3)	Anxiety & depression	No	30 modules (8 weeks)	- Text chapters with figures and images - Audio with every chapter - Cartoon videos	CBT^a^	- Worksheets - Symptom tracking - Diary
Changing States -The Stress and Anxiety Manager (#4)	Anxiety & stress	No	1 module divided in 4 main sections	- Slides with images, text, accompanied by audio - Notes for printing	Hypnotherapy & CBT^a^	- Relaxation technique audio files
CBT 7 Step Self Help Course (#5)	Anxiety, depression, & anger	Option of receiving paid email guidance and personalized formulation	7 modules	- Text chapters with figures - Audio features (need to be purchased separately)	CBT^a^	- PDF worksheet - Wiki
CCBT Limited – FearFighter (#6)	Panic and phobia	Via telephone (if purchased)	9 steps (recommended 9 weeks)	- Video text and image slides	CBT^a^	- Worksheet printouts - Progress monitoring - Emails with further tips at the end of each step
eCentreClinic - Mood Mechanic Course (#7)	Depression, social anxiety, panic attacks, & generalized worry; Australian adults aged 18 to 24	Weekly contact with clinician via email and telephone (depends on trial)	4 lessons (5 weeks)	- Text chapters and images	CBT^a^	- Online questionnaires and worksheets - Symptom tracking - Diary - Knowledge tests
eCouch - Anxiety & Worry Program (#8)	Anxiety & worry; Aged over 16	No	3 main sections (arm chair: 15 sections, tool kit, workbooks)	- Text chapters with figures and animated pictures - Audio features	CBT^a^& IPT^b^	- Online questionnaires and worksheets - Symptom tracking - Diary - Knowledge tests
eCouch - Social Anxiety Program (#9)	Social anxiety; Aged over 16	No	3 main sections (arm chair: 16 sections, tool kit, workbooks)	- Text chapters with figures and animated pictures - Audio features	CBT^a^& IPT^b^	- Online questionnaires and worksheets - Symptom tracking - Diary - Knowledge tests
Learn to Live (#10)	Social anxiety	No	8 lessons (8 weeks recommended)	- Animated slides with audio, images, and text - Videos - Slides require input from users	CBT^a^	- Online and printable worksheets - Forum - Symptom tracking - Online calendar - Questionnaires
Livanda - Free from Anxiety (#11)	Panic disorder, social phobia, & general anxiety	Through messaging system within the program (if paid for)	8-10 sections (12-15 weeks)	- Text chapters and slides - Audio features	CBT^a^	- Online worksheets - Symptom tracking
Living Life to the Full (#12)	Anxiety, stress, & life skills	User can designate a support practitioner	12 modules	- Text slides with figures - Audio with every slide	CBT^a^	- Alert emails for incomplete modules - Symptom tracking - PDF worksheets - Online books - Online questionnaires
Mental Health Online - Generalised Anxiety Disorder (#13)	Generalized anxiety disorder; Aged over 18	Weekly eTherapist emails, monitor progress, answer questions and provide support via email	12 modules (12 weeks)	- Text chapters with figures - Audio and video features	CBT^a^	- PDFs worksheets - Online worksheets - Symptom tracking - Diary
Mental Health Online - Social Anxiety Disorder (#14)	Social anxiety disorder; Aged over 18	Weekly eTherapist emails, monitor progress, answer questions and provide support via email	12 modules (12 weeks)	- Text chapters with figures - Audio and video features	CBT^a^	- PDF worksheets - Online worksheets - Symptom tracking - Diary
Mental Health Online - Panic Disorder with or without Agoraphobia (#15)	Panic disorder with or without agoraphobia; Aged over 18	Weekly eTherapist emails, monitor progress, answer questions and provide support via email	12 modules (12 weeks)	- Text chapters with figures - Audio and video features	CBT^a^	- PDF worksheets - Online worksheets - Symptom tracking - Diary
Mood Control (#16)	Anxiety & depression	No	12 modules (13 weeks)	- Video for every chapter with an introduction text	CBT^a^	- PDF worksheets - Online questionnaires - Symptom tracking - Forum - Bonus material (sessions for personal development and life change) - Additional worksheets
MoodGym (#17)	Anxiety & depression; Aged over 16	No	5 modules	- Text chapters with images	CBT^a^& IPT^b^	- Quizzes - Worksheets - Downloadable relaxation audio - Symptom tracking
myCompass (#18)	Anxiety, depression, & stress; Aged over 18; Mobile phone function for Australian residents only	No	12 modules (6-8 weeks)	- Text chapters with figures	CBT^a^, IPT^b^, & positive psychology	- PDF worksheets - Online worksheets - Symptom tracking - Diary - Wiki - SMS & email reminders - Real-life experience stories
Online Therapy – Anxiety (#19)	Anxiety; Aged over 18	Live support and email	8 sections (8 weeks)	- Text chapters with figures and images	CBT^a^	- Online worksheets - Online questionnaires - Symptom tracking - Diary - Forum - Chatroom for general help
Online Therapy - Generalized Anxiety Disorder (#20)	Generalized anxiety disorder; Aged over 18	Live support and email	8 sections (8 weeks)	- Text chapters with figures and images	CBT^a^	- Online worksheets - Online questionnaires - Symptom tracking - Diary - Forum - Chatroom for general help
Online Therapy - Panic Attacks (#21)	Panic attacks; Aged over 18	Live support and email	8 sections (8 weeks)	- Text chapters with figures and images	CBT^a^	- Online worksheets - Online questionnaires - Symptom tracking - Diary - Forum - Chatroom for general help
Online Therapy - Agoraphobia (#22)	Agoraphobia; Aged over 18	Live support and email	8 sections (8 weeks)	- Text chapters with figures and images	CBT^a^	- Online worksheets - Online questionnaires - Symptom tracking - Diary - Forum - Chatroom for general help
Online Therapy - Social Anxiety (#23)	Social anxiety; Aged over 18	Live support and email	8 sections (8 weeks)	- Text chapters with figures and images	CBT^a^	- Online worksheets - Online questionnaires - Symptom tracking - Diary - Forum - Chatroom for general help
Online Therapy - Speech Anxiety (#24)	Speech anxiety; Aged over 18	Live support and email	8 sections (8 weeks)	- Text chapters with figures and images	CBT^a^	- Online worksheets - Online questionnaires - Symptom tracking - Diary - Forum - Chatroom for general help
Serenity Program - Anxiety Program (#25)	Stress, generalized anxiety disorder, social anxiety, & panic disorder; Aged over 18	No	9 modules (9 weeks)	- Text slides with figures and animated images - Interactive content on slides - Audio feature	CBT^a^	- PDF worksheets
Social Anxiety Institute (#26)	Social anxiety	No	25 modules	- Audio sessions - Video features - Hand-outs	CBT^a^	None
This Way Up Clinic – Worry (#27)	Generalized anxiety disorder; Aged over 18	Supervised by clinician	6 modules (8 weeks)	- Comic slides	CBT^a^	- Online questionnaires - Downloadable homework - Symptom tracking - Recovery stories - Online calendar (set up email reminders) - Downloadable extra activities and information
This Way Up Clinic -Worry and sadness (#28)	Depression & anxiety; Aged over 18	Supervised by clinician	6 modules (8 weeks)	- Comic slides	CBT^a^	- Online questionnaires - Downloadable homework - Symptom tracking - Recovery stories - Online calendar (set up email reminders) - Downloadable extra activities and information
This Way Up Clinic – Panic (#29)	Panic/agoraphobia; Aged over 18	Supervised by clinician	6 modules (8 weeks)	- Comic slides	CBT^a^	- Online questionnaires - Downloadable homework - Symptom tracking - Recovery stories - Online calendar (set up email reminders) - Downloadable extra activities and information
This Way Up Clinic - Shyness (#30)	Social phobia; Aged over 18	Supervised by clinician	6 modules (8 weeks)	- Comic slides	CBT^a^	- Online questionnaires - Downloadable homework - Symptom tracking - Recovery stories - Online calendar (set up email reminders) - Downloadable extra activities and information
This Way Up Self-help - Shyness (#31)	Social phobia; Aged over 18	No	3 modules (3 weeks)	- Comic slides	CBT^a^	- Online questionnaires - Downloadable homework - Symptom tracking - Recovery stories - Online calendar (set up email reminders) - Downloadable extra activities and information
This Way Up Self-help - Worry and Sadness (#32)	Depression & anxiety; Aged over 18	No	3 modules (3 weeks)	- Comic slides	CBT^a^	- Online questionnaires - Downloadable homework - Symptom tracking - Recovery stories - Online calendar (set up email reminders) - Downloadable extra activities and information
This Way Up School -Overcoming Social Anxiety (#33)	Social anxiety; grade 11 and 12 high school	No	6 modules (6 weeks)	- Comic slides	CBT^a^	- Online questionnaires - Downloadable homework - Symptom tracking - Recovery stories - Online calendar (set up email reminders) - Downloadable extra activities and information
This Way Up School -Anxiety and Depression Prevention for Adolescents (#34)	Anxiety & depression; grade 9 to 11 high school	No	6 modules (6 weeks)	- Comic slides	CBT^a^	- Online questionnaires - Downloadable homework - Symptom tracking - Recovery stories - Online calendar (set up email reminders) - Downloadable extra activities and information

^a^CBT: Cognitive behavioral therapy

^b^IPT: Interpersonal Therapy

#### Intervention Focus

**Figure 4:**
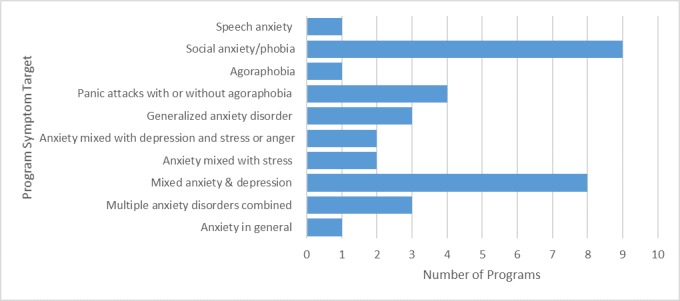
Intervention target of evaluated programs.

Programs were designed for a range of issues including specific anxiety disorders; anxiety combined with depression and stress, or anger; various anxiety disorders combined; or anxiety in general. Figure 4 shows that the majority of programs were designed for social anxiety/phobia (9/34, 28%) or for mixed anxiety and depression (8/34, 24%). The remaining programs focused on anxiety in general (1/34, 3%); multiple anxiety disorders combined (3/34, 3%); anxiety mixed with stress (2/34, 6%); anxiety mixed with depression and stress or anger (2/34, 6%); or other specific anxiety disorders such as generalized anxiety disorder (3/34, 9%), panic attacks with or without agoraphobia (4/34, 12%), agoraphobia (1/34, 3%), and speech anxiety (1/34, 3%). Concerning the target audience, the majority of programs were designed for an adult population (aged over 16 or 18 years) (20/34, 59%), 2 were targeted at teenagers of high school age, 1 specifically for young adults aged 18-24 years, and 11 programs (32%) did not specify an age group, but based on content seemed to be designed for adults.

#### Intervention Design

**Figure 5:**
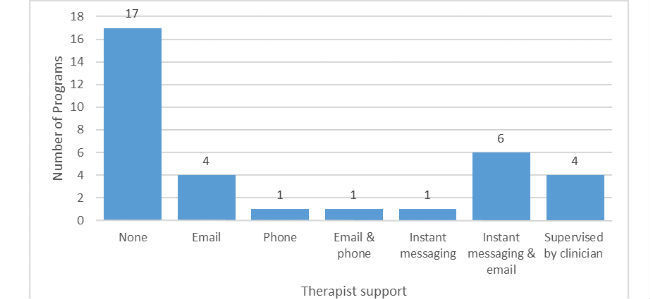
Therapist support offered in evaluated programs.

In total, 17 programs (50%) offered therapist support, either by email, instant messaging, or phone. See Figure 5 for a summary of the different forms of therapist support. Therapist support always required a fee; for the majority of these support programs (10/17, 59%), there was an option of paying only for the self-guided version or paying extra for support. For one free program (Living Life to the Full), consumers could invite a professional to access their account and provide support within the program (support practitioner). The recommended length of the programs varied from 1 to 15 weeks (mean 8.85 weeks, SD 4.10) and the number of modules offered ranged from 1 to 30 (mean 9.38, SD 5.97).

#### Modes of Therapy Presentation

All programs used a combination of different modes of presentation (eg, text, images, audio, video, text entry-fields, and animation). Content was most frequently presented as text chapters with images or diagrams (23/34; 68%). Other identified modes of presentation included animated slides or pictures, comic slides, ebooks, and video sessions. In total, 15 programs (44%) incorporated audio components and 9 included video components (26%).

#### General Therapeutic Approach

**Figure 6:**
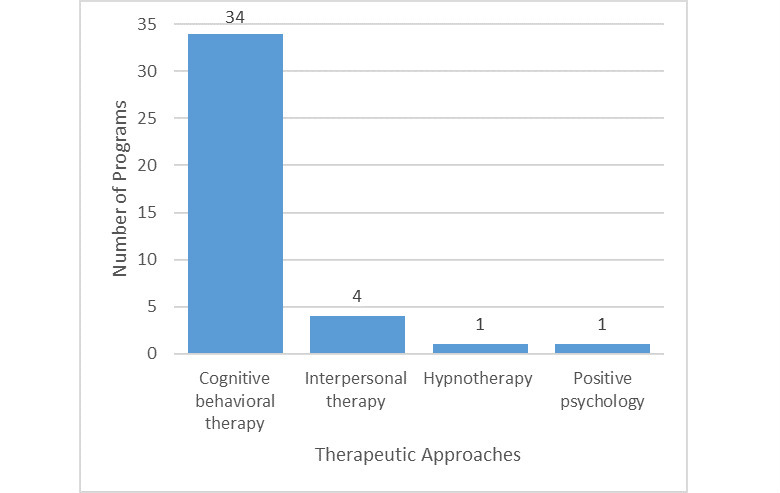
Therapeutic approaches used in evaluated programs.

All 34 programs claimed to be CBT-based and at least one cognitive and behavioral therapeutic element was employed for each program based on the examined module content. Some programs stated that they also incorporated other therapeutic approaches, such as interpersonal therapy (4/34, 13%), hypnotherapy (1/34, 3%), and positive psychology (1/34, 3%) (see Figure 6).

#### Other Therapeutic Elements

Other popular therapeutic elements included psychoeducation modules, goal-setting features, features to create case conceptualizations for oneself, skills training exercises, various forms of relaxation exercises, mindfulness-based exercises, experience stories, sleep hygiene, and relapse prevention.

#### Intervention Features

All except for one program (33/34, 97%) provided the user with worksheets during the session or homework in PDF or online forms. Mood or symptom monitoring/tracking was part of the majority of programs (29/34, 85%). Most programs allowed the user to see results and access a result history either in a numerical or diagram format. In total, 12/34 programs (35%) offered an online diary and 9/34 programs (27%) incorporated a user forum. The review also revealed a great variety of other program features. One feature was the set-up of email or text message reminders for unfinished or future sessions (AI-Therapy, Beating the Blues, Living Life to the Full, myCompass) and an online treatment calendar to schedule the next session and set up alerts (Learn to Live, This Way Up programs). Other features included bonus material (eg, personal development offered in Mood Control), additional resources (ie, more worksheets to be used between sessions or after the end of treatment, offered in Mood Control, This Way Up), treatment items voted most useful by users, to-do-list maker, personal note section, awards, commitment checks (Blues Begone), knowledge tests at the beginning and information about medication (AI-Therapy, e-couch), personalized eBooks (AI-Therapy), printable session recap and homework cards in wallet format (Mental Health Online), and a teammate function, which allowed the nomination of friends or family members for optional support (Learn to Live).

### Empirical Evidence for Program Efficacy

A summary of the types of published research evaluations for each program and the respective references can be found in Table 4. For 3/34 programs (9%), indirect research evidence was identified. The two e-couch programs are based on the MoodGYM program, for which research evidence is available; however, the e-couch program’s efficacy was not specifically empirically evaluated. The AI-Therapy program has only been evaluated for social anxiety in adults who stutter using a pre-post study without a control group. For 17/34 programs (50%), empirical studies evaluating efficacy or effectiveness were found. Studies ranged from case series and small to mid-sized pre-post interventions without comparison groups to controlled and randomized controlled trials (RCT). Both MoodGYM and This Way Up have been evaluated through 9 RCTs each. The efficacy of Beating the Blues was demonstrated by 2 RCTs, FearFighter by 2 RCTs, and myCompass by 1 RCT. For 14/34 programs (41%), no research evidence of the efficacy or effectiveness of the intervention was found.

**Table 4:** Types of research evaluations of included Web-based interventions

Program (Ref#)	Type of Research Evaluation Studies
AI-Therapy (#1)	- Pre-post intervention for social anxiety in adults who stutter [42] - Case study [43]
Beating the Blues (#2)	- Feasibility & acceptability [44] - 2 RCTs^a^[45, 46] - Cost-effectiveness [47] - Pre-post intervention without comparison group [42, 49] - Implementation [50- 52]
Blues Begone (#3)	- Pre-post intervention without comparison group [53]
Changing States - The Stress and Anxiety Manager (#4)	Website: not specified; Beacon^b^: no research evidence
CBT 7 Step Self Help Course (#5)	Website: not specified; Beacon^b^: not reviewed
CCBT Limited – FearFighter (#6)	- Acceptability study [54] - Pre-post intervention pilot [55] - Case studies without comparison group [56] - Implementation study [57] - 2 RCTs^a^[58, 59]
eCentreClinic - Mood Mechanic Course (#7)	Website: nothing for this specific program; Beacon^b^: not reviewed
eCouch - Anxiety & Worry Program (#8, #9)	Adapted from MoodGYM
Learn to Live (#10)	Website: not specified; Beacon^b^: not reviewed
Livanda - Free from Anxiety (#11)	Website: not specified; Beacon^b^: no research evidence
Living Life to the Full (#12)	Website: not specified; Beacon^b^: no research evidence
Mental Health Online - Generalized Anxiety Disorder (#13, #14, #15)	- Participant choice trial [60] - Implementation [61] - Predictors of pre-treatment attrition and treatment withdrawal [62]
Mood Control (#16)	Website: not specified; Beacon^b^: no research evidence
MoodGym (#17)	- Nine RCTs^a^ [63- 71] - School and class-based trials [72, 73] - Implementation [74] - Program usage analysis [75] - Follow-up outcome analysis [76] - Compliance of community users and predictor of outcomes analysis [77]
myCompass (#18)	- RCT^a^[78, 79]
Online Therapy (#19, #20, #21, #22, #23, #24)	Website: not specified; Beacon^b^: not reviewed
Serenity Program - Anxiety Program (#25)	- Pilot pre-post treatment without comparison group [80]
Social Anxiety Institute (#26)	Website: not specified; Beacon^b^: not reviewed
This Way Up Clinic (#27, #28, #29, #30) This Way Up Self-help (#31, #32)	Generalized anxiety disorder: - 3 RCTs^a^[81- 83] - Implementation study [84]
This Way Up School (#33, #34) Anxiety	Panic: - 1 RCT^a^[85] - Pre-post intervention trial without comparison group [86] Social phobia: - 5 RCTs^a^[87- 91] - Implementation study [92] - Cost-effectiveness, acceptability, and follow-up analysis [93]

^a^RCT: randomized controlled trial

^b^Beacon: Australian clinical online platform that describes different Web-based self-help treatment programs [35]

Results were examined for programs for which anxiety symptoms were evaluated in RCTs. Beating the Blues was found to lead to a significant reduction of anxiety both at the end of treatment and at 6 months’ follow-up compared to treatment as usual [45]. For FearFighter, both the face-to-face and online program group had reduced anxiety at post-treatment and 1-month follow-up [59]. MoodGym was evaluated in adolescents and university students and levels of anxiety were found to be lower in the intervention group compared to the waitlist control group after the intervention [63,66]. In addition, combined face-to-face and online CBT was more effective in treating anxiety symptoms than either face-to-face or online as a standalone [70]. Compared to control subjects, participants in the myCompass intervention had reduced anxiety symptoms after the program and symptom scores remained at near normal levels at 3-month follow-up [78]. For This Way Up, a program targeted at generalized anxiety disorder, the intervention group participants showed significantly reduced symptoms of panic [81] and anxiety [82] at post-treatment compared to the control group. Symptom reduction was the same for technician and clinician-assisted versions of the treatment [83].

### Program Evaluation

Program evaluation scores for each program and each evaluation criteria can be found in Multimedia Appendix 3. Scores for each program ranged from 69% (CBT 7 Step Self Help Course) to 100% (AI-Therapy) with an average score of 81% (SD 7%). Concerning the evaluation criteria, all program websites specified for which patient group or symptoms the program was designed, defined or stated the utilized model of change, presented program author names and credentials, and provided contact details. About half of the program websites had been empirically evaluated (19/34, 55.9%), specified which information was covered in the intervention modules (18/34, 52.9%), and provided evidence for the program to the user (eg, attrition data, success rate, completion rate, number of users in the program, testimonials) (14/34, 41.2%). Only 4 program websites (11.8%) specified whether the intervention was tailored to the user or generic for all users. This question could not be evaluated for one website (Social Anxiety Institute).

## Discussion

### Principal Findings

To our knowledge, this is the first review of publically available Web-based programs for anxiety that showcases what individuals seeking such treatment options might find if they search the Web. The review aimed at providing consumers, practitioners, and researchers with a summary of the availability, characteristics, and efficacy of currently freely available Web-based interventions for anxiety. The review identified a wide variety of programs for anxiety, specific anxiety disorders, or anxiety in combination with stress, depression, or anger with treatments based predominantly on CBT techniques. The majority of websites were found to be credible and accessible. Of the programs reviewed, the majority required that users register and/or pay a program access fee. Half of the programs offered some form of paid therapist or professional support. Programs varied in treatment length and number of modules and employed a variety of presentation modes. Relatively few were evaluated in terms of efficacy. In particular, this review highlights two key issues: the large number and diversity of program formats and the lack of empirical evidence of efficacy for many of the identified programs. These will be discussed in more detail and results will be compared with a similar review of Web-based depression programs available on the Web [37].

First, the great variety and large number of identified programs for anxiety is noteworthy. Programs differed in their level of support, accessibility, and presentation. A similarly great variability among identified programs was also found for Web-based depression interventions [37]. Concerning accessibility, more than half of the programs required an access fee. Considering the high costs and waiting times for psychotherapy in many countries, paid Web-based programs may provide an affordable alternative. However, programs often could only be purchased for a limited period. Many users may not be able to finish the program in the allotted time, and being able to receive treatment at one’s own pace might be an important reason for choosing Web-based treatment over face-to-face therapy.

Overall, most programs used a multimedia presentation for the intervention delivery. With the current rapid pace of advances in technology, more engaging ways of translating therapeutic techniques into interactive techniques could be created for Web-based interventions to distinguish them from traditional self-help material. Increased engagement through interactivity may increase adherence and effectiveness [94,95], especially when considering reports of low utilization and high dropout rates of Web-based interventions [19,96]. As individuals may differ in their preferred style of therapy and time and resources available for treatment, trying different programs and considering the access period is recommended before choosing a program.

The number of identified anxiety programs was similar to the number listed in the Beacon directory [35]. In total, 33 distinct programs were found in the directory. About half of those programs were also identified by this review and some of those identified in this review were not listed on Beacon. It is important to note here that the Beacon website is not updated very often; for example, some reviews of anxiety programs were last updated in 2009. The difference may also be a result of the keywords used and the way search engines are designed and work. Search engines are often referred to as “information gatekeepers,” as they are able to include and exclude websites and influence the ranking of websites in the search results [39]. These results suggest that even though a multitude of Web-based programs exists, it may be difficult for interested consumers to identify and compare all options. Having specialized services like the Beacon directory and keeping them up-to-date is therefore important to provide consumers with knowledge about program differences, credibility, and effectiveness. This will in turn help consumers to be able to compare programs and choose the one most suitable for them. A review of Web-based depression interventions identified a similar number (n=32) of programs on the Web and 12 of those programs were also included in this review [37]. Those were mostly programs that offered interventions targeting both anxiety and depression issues.

To ensure that consumers access programs of appropriate quality and safety, national and/or international platforms are needed that provide consumers with reliable guidance on evidence-based and effective Web-based intervention options. For example, the E‐Mental Health Strategy for Australia [97] outlines the development of an e-mental health portal that provides reliable information and accessible pathways for consumers and caregivers to navigate and use evidence-based Web-based mental health support. In addition, there is little consistent guidance on necessary quality standards, as well as legal and ethical issues regarding Web-based interventions for professionals. There is, for example, the Suggested Principles for the Online Provision of Mental Health Services by the International Society for Mental Health Online (ISMHO); however, this document mainly addresses online counselling and there are no guidelines specifically addressing Web-based programs. In the context of mental health apps, a review has also highlighted the need for standards and guidelines for developers to follow and frameworks for consumers to assess credibility and legitimacy [32].

Concerning the evidence base of the included programs, all were found to be based on CBT principles. This is consistent with prior reviews, which found that some form of CBT or other behavioral therapy was included in most Web-based interventions [98], as well as in publically available Web-based intervention programs for depression [37]. In general, research evidence indicates that CBT is an effective treatment for anxiety disorders (eg, [99,100]. This suggests that all reviewed programs were to some extent developed using an evidence-based approach; however, this does not guarantee that the evidence-based approach used is necessarily effective in the program.

In this context, another major finding was that several programs did not provide any research evidence or provided only limited evidence of the efficacy of the treatment. This is similar to findings from the review of Web-based depression interventions, which showed that 63% had not been evaluated using RCTs [37]. This finding is interesting considering the numerous systematic reviews and meta-analyses of Web-based interventions [25,30,101]. However, some may currently be in the process of being evaluated and not yet published. In addition, the absence of evidence of efficacy in terms of RCTs for a particular intervention also does not necessarily mean that the intervention does not work, especially if it is based on evidence-based approaches such as CBT. For treatment efficacy, the predominant model has been “empirically supported treatments” [102]. However, recently it has been proposed that clinical treatment decisions should be based on the best available research evidence, a clinician’s expertise, and patient characteristics [103]. It has also been argued that RCTs evaluating interventions should focus on evaluating intervention principles rather than each actual implementation [104]. However, unlike therapists who require accreditation to practice an evidence-based approach such as CBT, no such accreditations currently exist for Web-based programs. Hence, any Web-based program can claim to be based on CBT, but may not fulfil all requirements and therefore not work, which is especially problematic for programs requiring an access fee. Therefore, programs should ideally undergo appropriate empirical evaluation before being made available online. The development of an accreditation service for Web-based interventions may help improve this issue and enable consumers to make more informed decisions. It is also important to acknowledge competing interests within the eHealth space. Developers with a commercial focus may not be as concerned about treatment efficacy and researchers developing programs may not have the resources to sustain a publically available program. For programs for which published empirical studies were identified in this review, there was a large variety of study designs and quality of evidence. Only This Way Up programs, MoodGYM, myCompass, Beating the Blues, and FearFighter underwent rigorous evaluation through RCTs.

### Limitations

In regard to this study, a few limitations have to be noted. The representativeness and comprehensiveness of the search and identified programs may be affected by various characteristics of the Web, search engines, and search terms. First, the ranks of websites vary by location on commercial search engines. The search for this review was performed in the UK and it is likely that the same search in another country may have yielded different results. The Web and search engines are also dynamic. Results of search engines vary over time, meaning searches conducted several months before or after the current search could present a different set of programs. In addition, currently existing programs may change or be discontinued and new programs may be released. It is also possible that some individuals may not use the three search engines and would have therefore received different results. However, a considerable strength of this review is that the three most popular search engines were used.

Secondly, the first 25 hyperlinks from the search were included in this review. It is likely that more programs are available, which at the time did not have the page ranking to be identified by the search. This may especially be the case for recently created services [105]. However, it has been suggested that most people rarely consider more than the first 20 links [40]; thus the identified sample of this review is believed to be representative of what an average Internet user might discover when searching the Web for Web-based intervention options. Page ranking is also influenced by various search engine optimization techniques, algorithms of the search engines, as well as cookie settings of the browsers [106], and thereby impacts the results of the search. To combat this, we removed search engine histories and cookies were disabled on all three browsers. While this may not be a complete list of currently available Web-based programs for anxiety, it is a comprehensive snapshot of programs found in March 2015.

Third, the program evaluation scale used was an adapted version of the scoring system used by Renton et al [37]. However, the summative scores do not account for the fact that items within this scale may not be equivalent in terms of importance. Using different weightings based on importance would add great value to the rating. The development of such a scale was considered beyond the scope of this review; however, it would be important to develop a weighted scale for similar future reviews.

Lastly, it is important to acknowledge that the definition of Web credibility is complex and consists of multiple dimensions [107]. In the case of this paper, only a limited number of credibility dimensions that focused on trustworthiness rather than expertise, were assessed.

### Conclusion

This review found that individuals searching for Web-based intervention programs for anxiety are presented with a large number and variety of potential programs to choose from. For consumers with limited knowledge about intervention quality criteria it may be challenging to choose an appropriate program. With the number of people using the Internet increasing, it is likely that more individuals will search for information about treatments options in general and, specifically, online interventions. It is therefore important for health professionals working with mental health clients to be aware of the diversity of Web-based interventions and that not all have had their efficacy tested in robust research trials. Directories such as Beacon can assist clinicians, as well as individuals in this task; however, it is important to keep services like these up-to-date. There is a definite need for consistent guidelines and standards on developing and providing Web-based mental health intervention programs for professionals and a platform with reliable up-to-date information for professionals and consumers about the quality and accessibility of Web-based interventions. This review is the first to identify and review Web-based anxiety interventions available on the Web. Therefore, research is needed in reviewing and evaluating Web-based intervention programs for other mental health related issues. There is also a need to develop standardized evaluation scales for publically available Web-based intervention programs to facilitate the rating process and ensure its rigor. For future research, it may also be interesting to explore health professionals’ and consumers’ experiences and perceptions of those programs.

## Appendix

Multimedia Appendix 1 Search log.

Multimedia Appendix 2 Screenshots of programs.

Multimedia Appendix 3 Program evaluation scores.

## Abbreviations

CBT: Cognitive behavioral therapy
IPT: Interpersonal Therapy
RCT: Randomized controlled trial
GP: General Practitioner
