# Supplementary material for: Finding Web-Based Anxiety Interventions on the World Wide Web: A Scoping Review
Source: JMIR Ment Health. 2016 Jun 1;3(2):e14. doi: 10.2196/mental.5349 (PMC4936762; doi:10.2196/mental.5349)

AI-Therapy (#1)

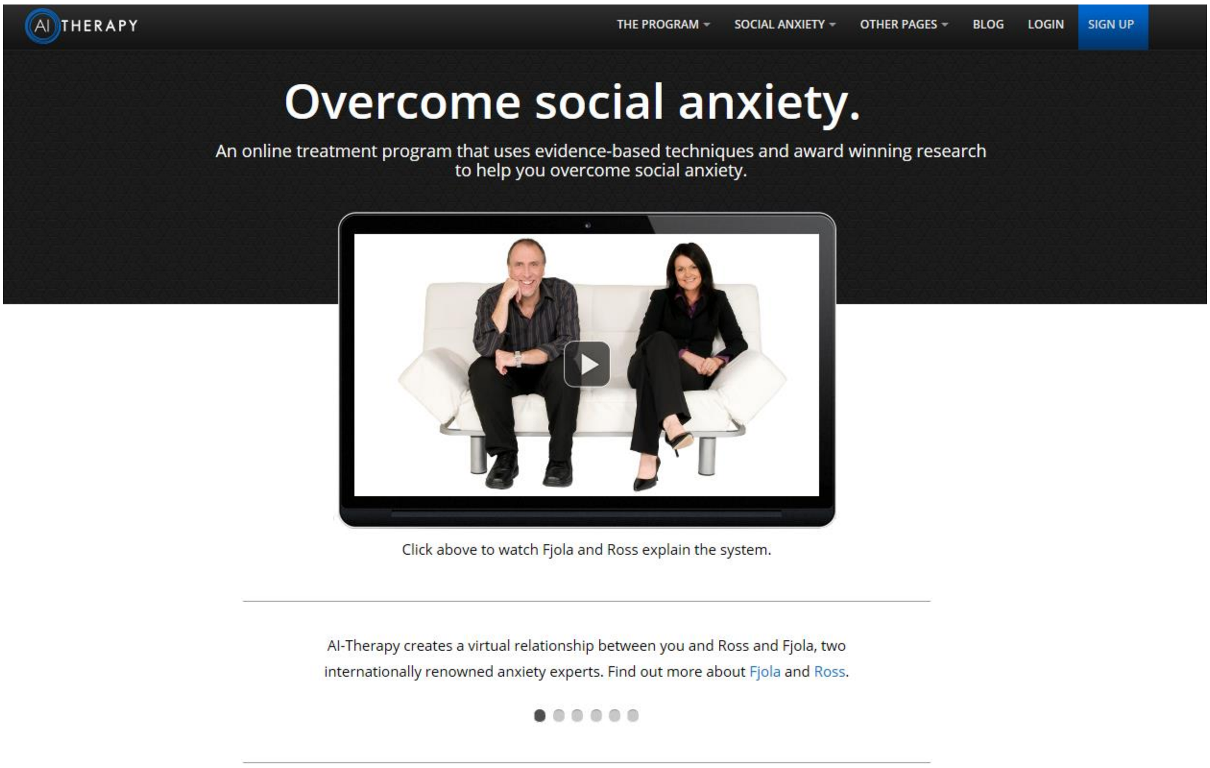

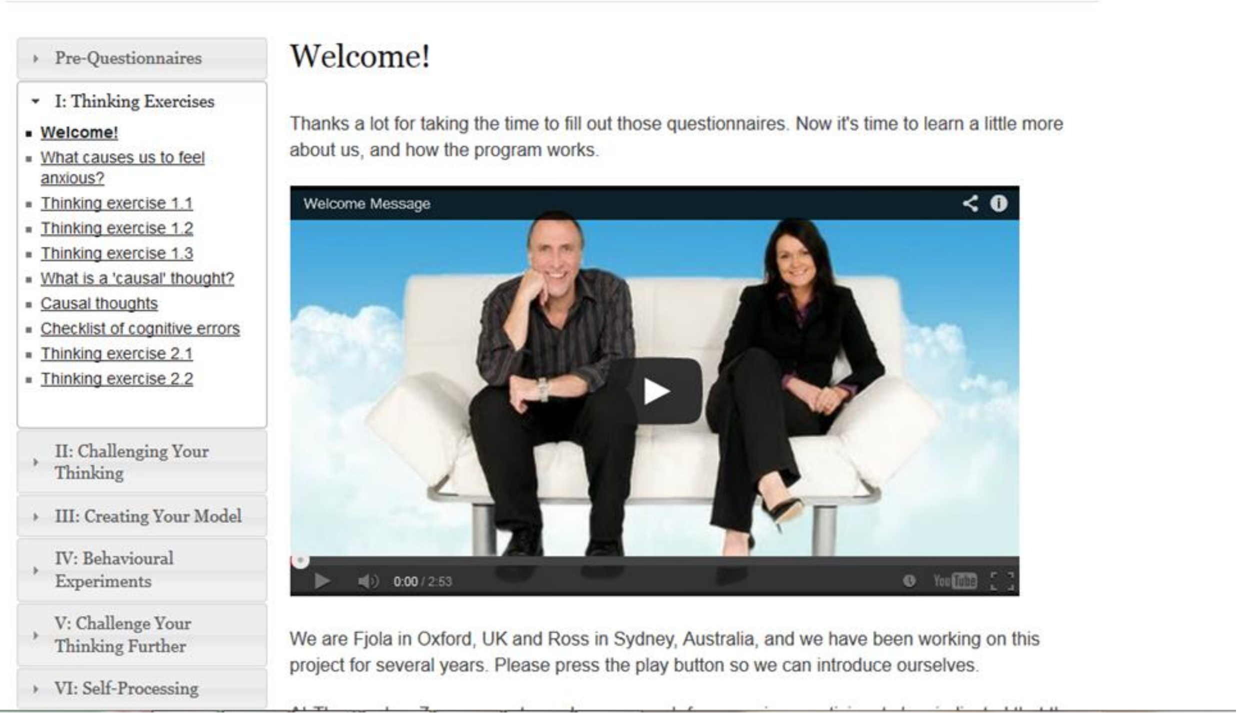

Beating the Blues (#2)

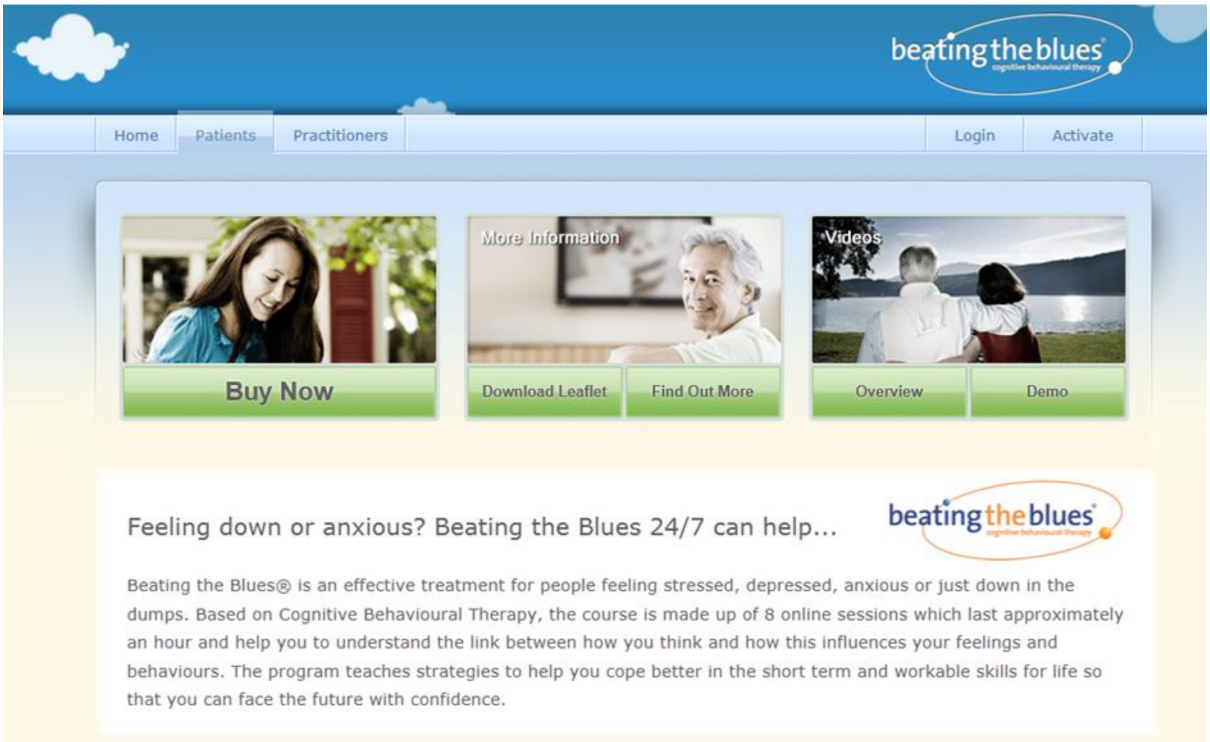

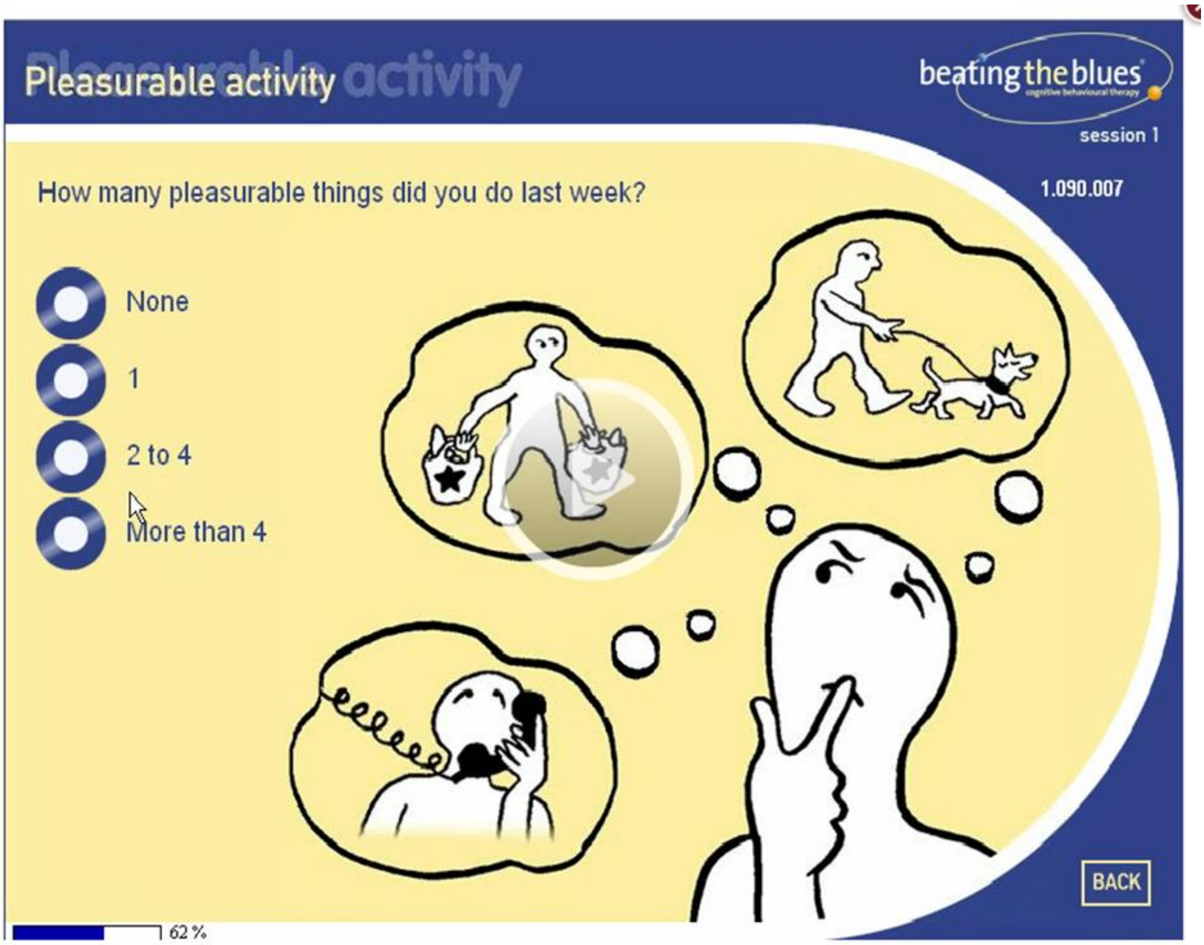

This video is a recording of a patient going through session 1 and learning about pleasurable events.

Download: 12MB

Blues Begone (#3)

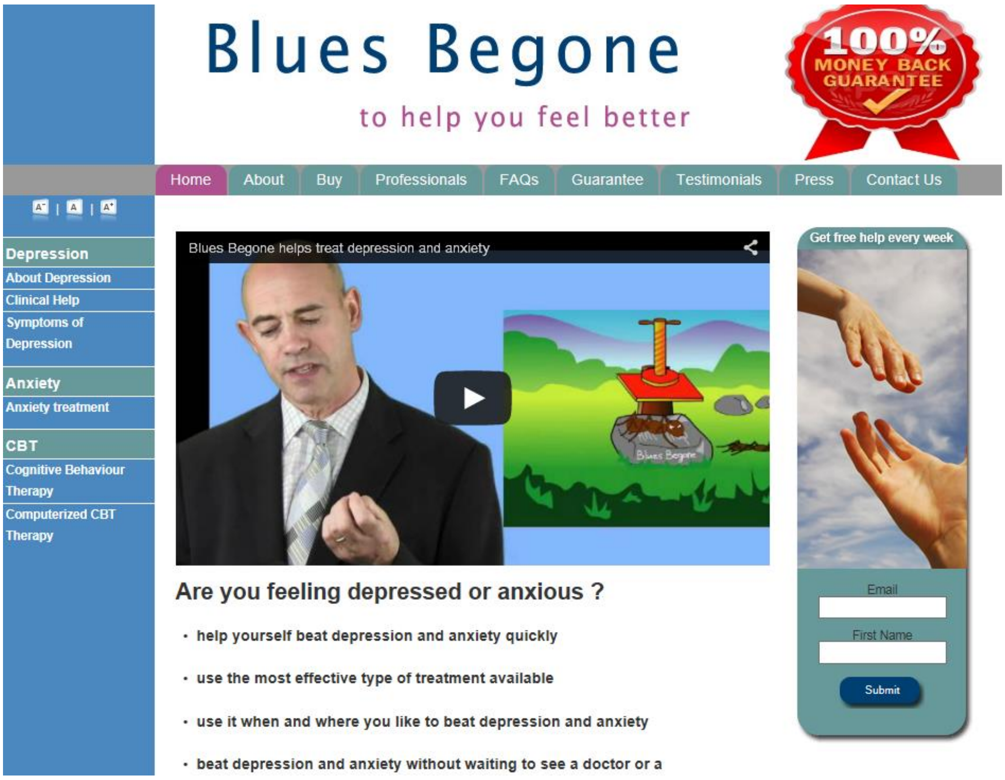

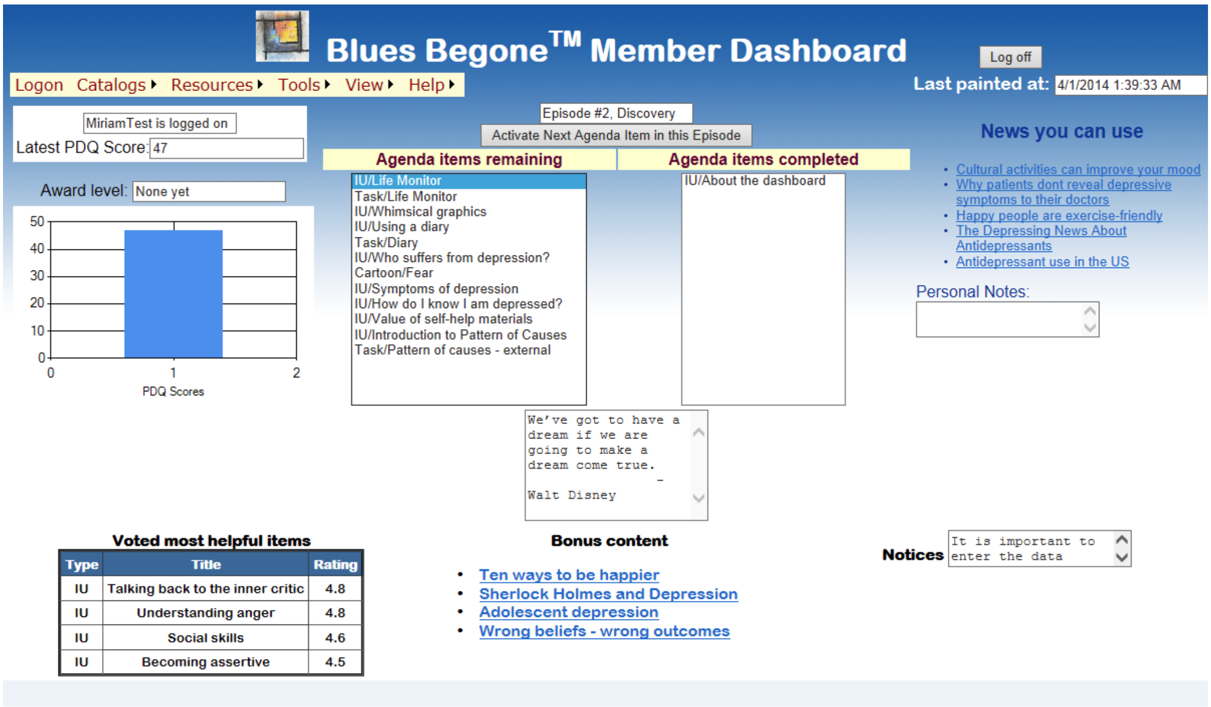

Changing States - The Stress and Anxiety Manager (#4)

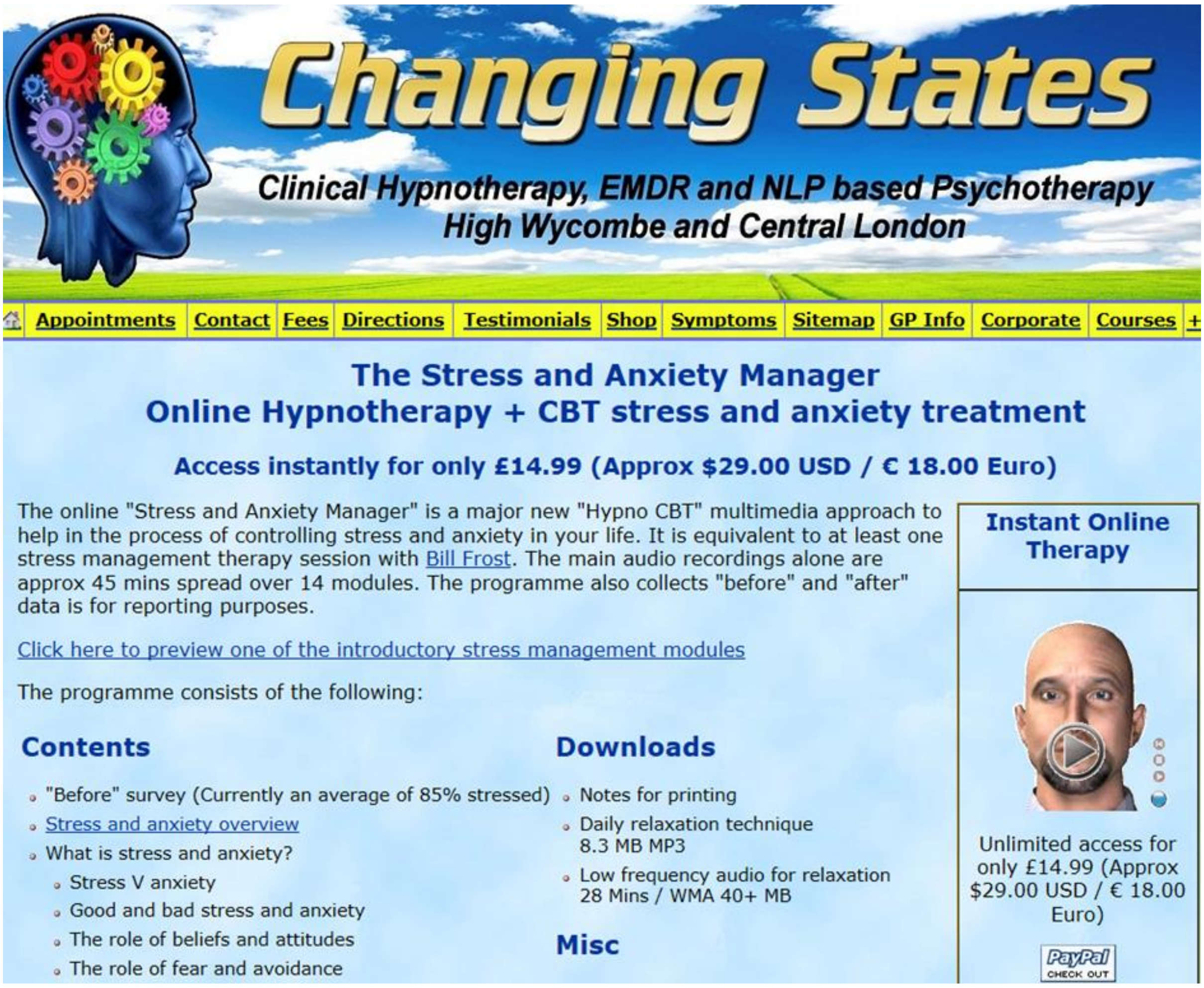

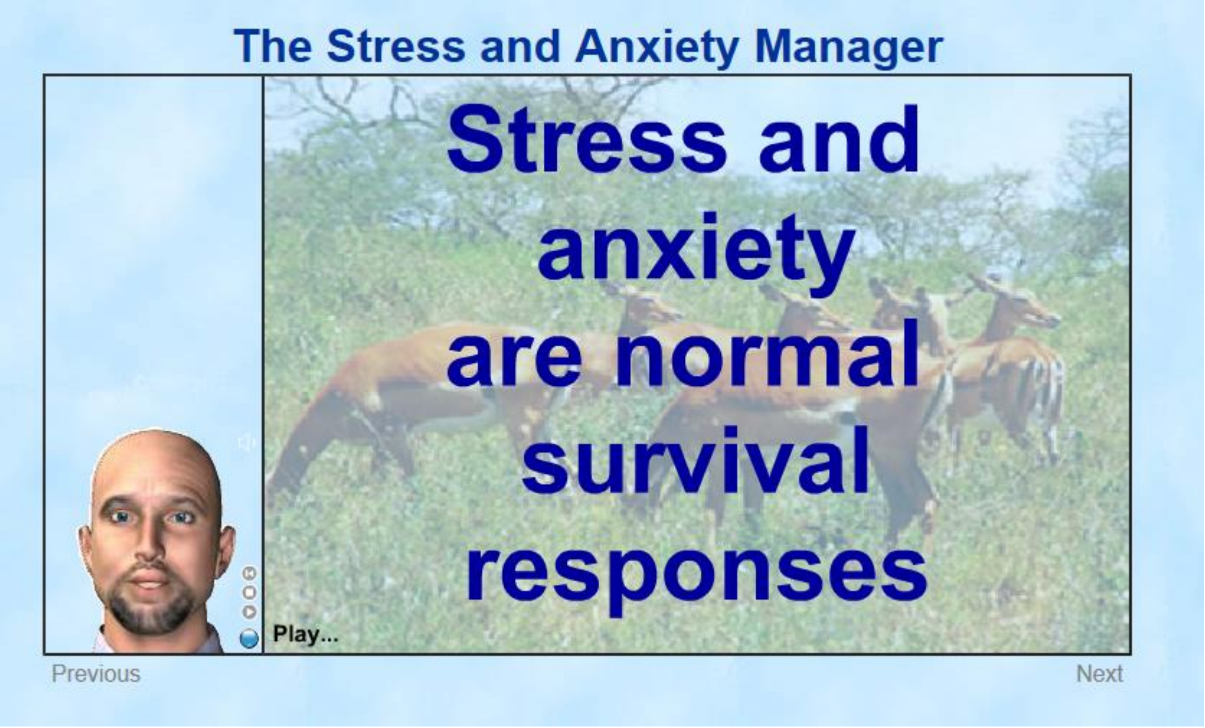

CBT 7 Step Self Help Course (#5)

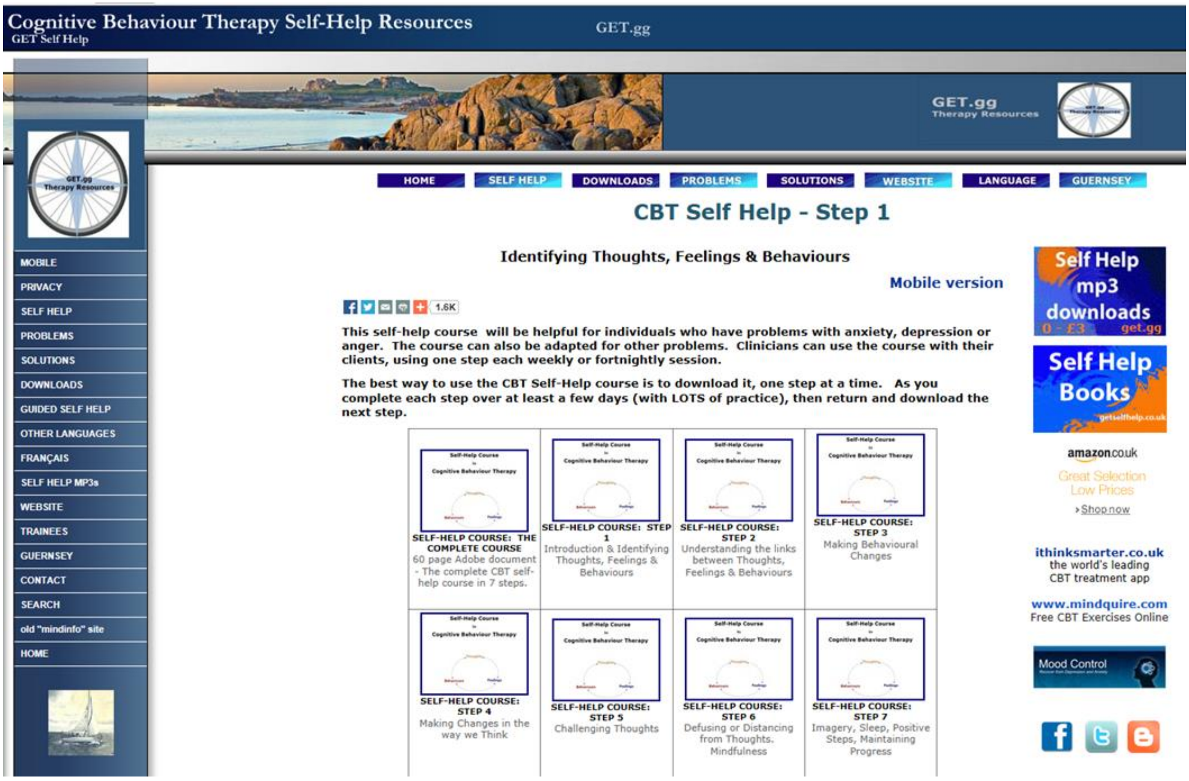

Step 1 Step 2 Step 3 Step 4 Step 5 Step 6 Step 7

The documents linked from the bottom of each page are intended to be an integral part of this course, and should not be omitted - the worksheet documents are included in the downloadable Steps linked above.

Please click here if you would like additional support in completing any of the steps of the CBT Self-Help Course

This mini 7-step self-help course includes techniques which could be useful for all, but professional help should be sought for complex or long-standing problems.

Use the Self Help mp3 downloads to strengthen therapy or as a stand-alone technique

Once you've read through the introduction to CBT on this page, you can start to look at what's helping to keep your problems going.

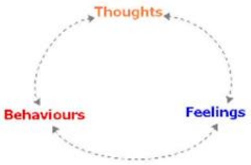

Let's use a recent example, when you've been distressed - perhaps something has happened in the past week or so when you've felt particularly anxious, angry, or depressed.

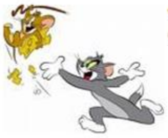

What was the situation? What happened? When did it happen? What else was going on? Was it a situation that you often find yourself in? Who were you with?

What thoughts or images went through your mind just before or during that time? If you had that thought, what did that mean to you, or what did that say about you or the situation? If your thought was a question, try to answer it. What disturbed me?

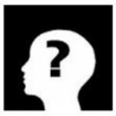

CCBT Limited – FearFighter (#6)

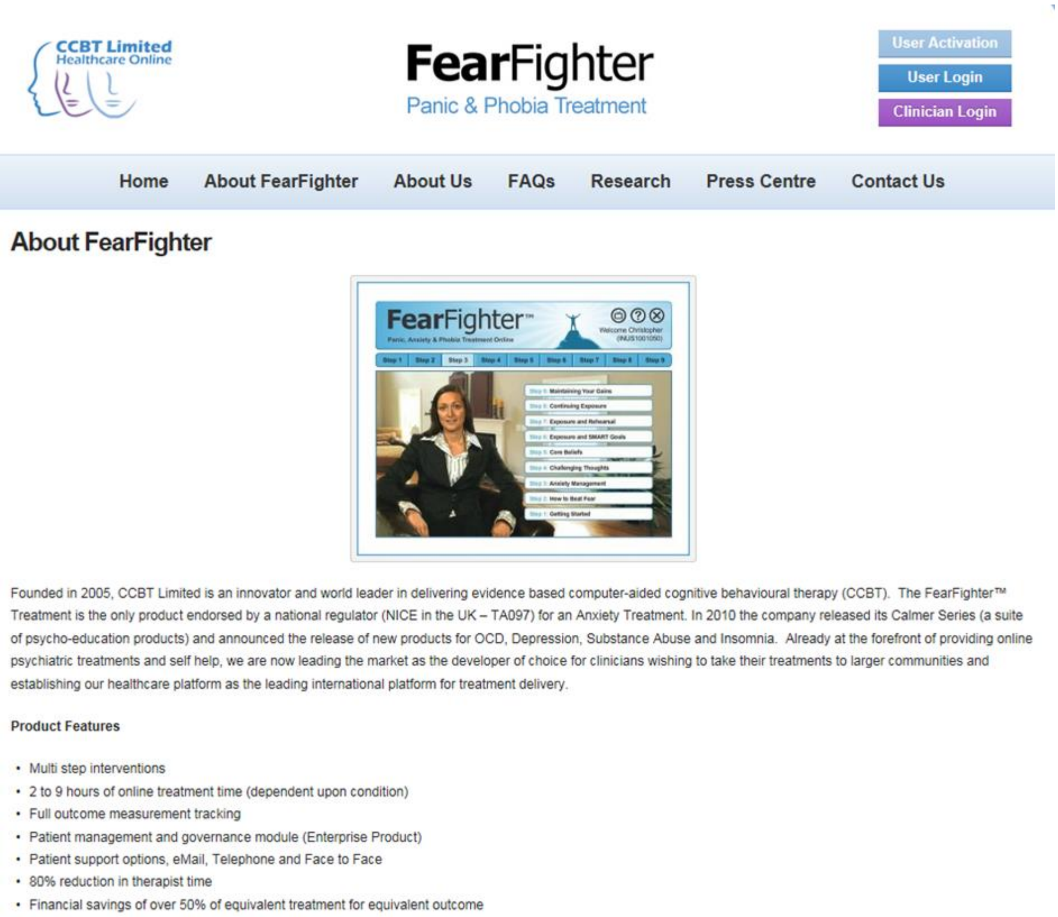

eCentreClinic - Mood Mechanic Course (#7)

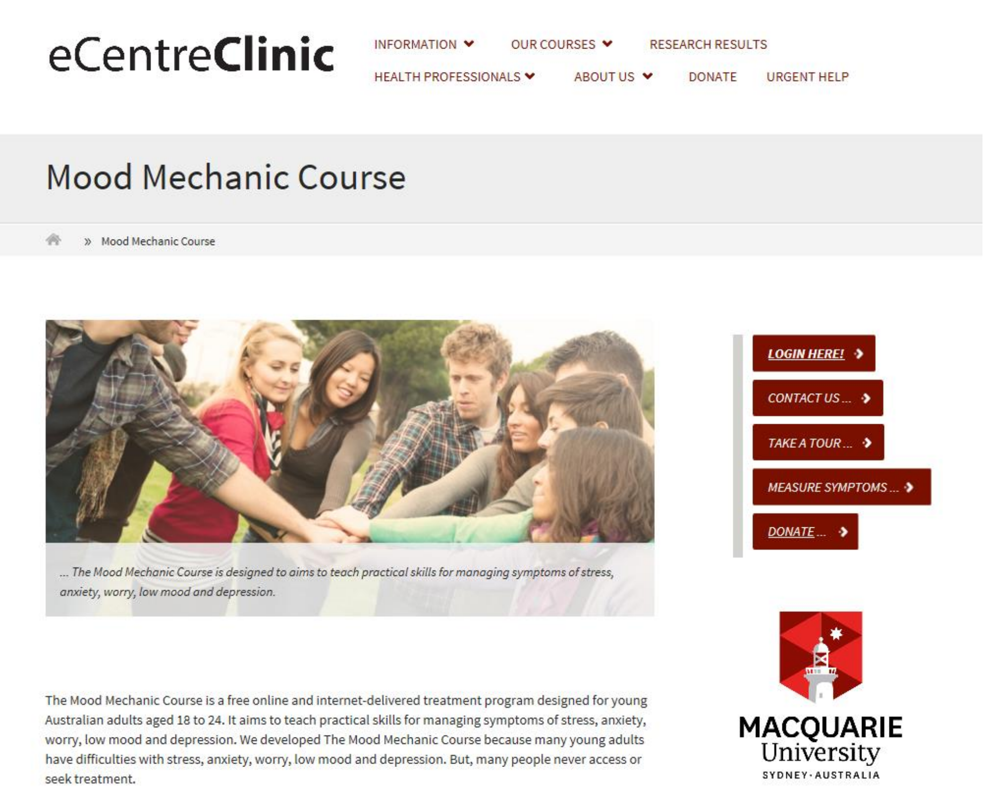

eCouch - Anxiety & Worry Program (#8, #9)

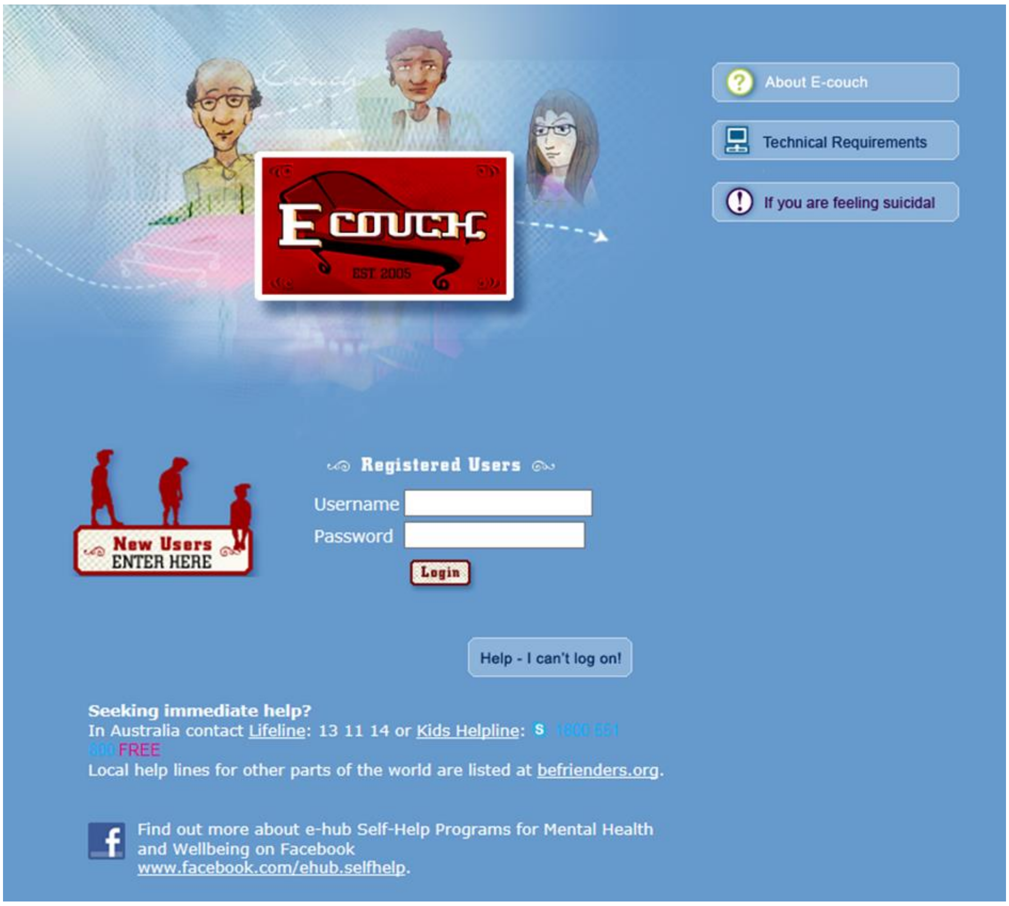

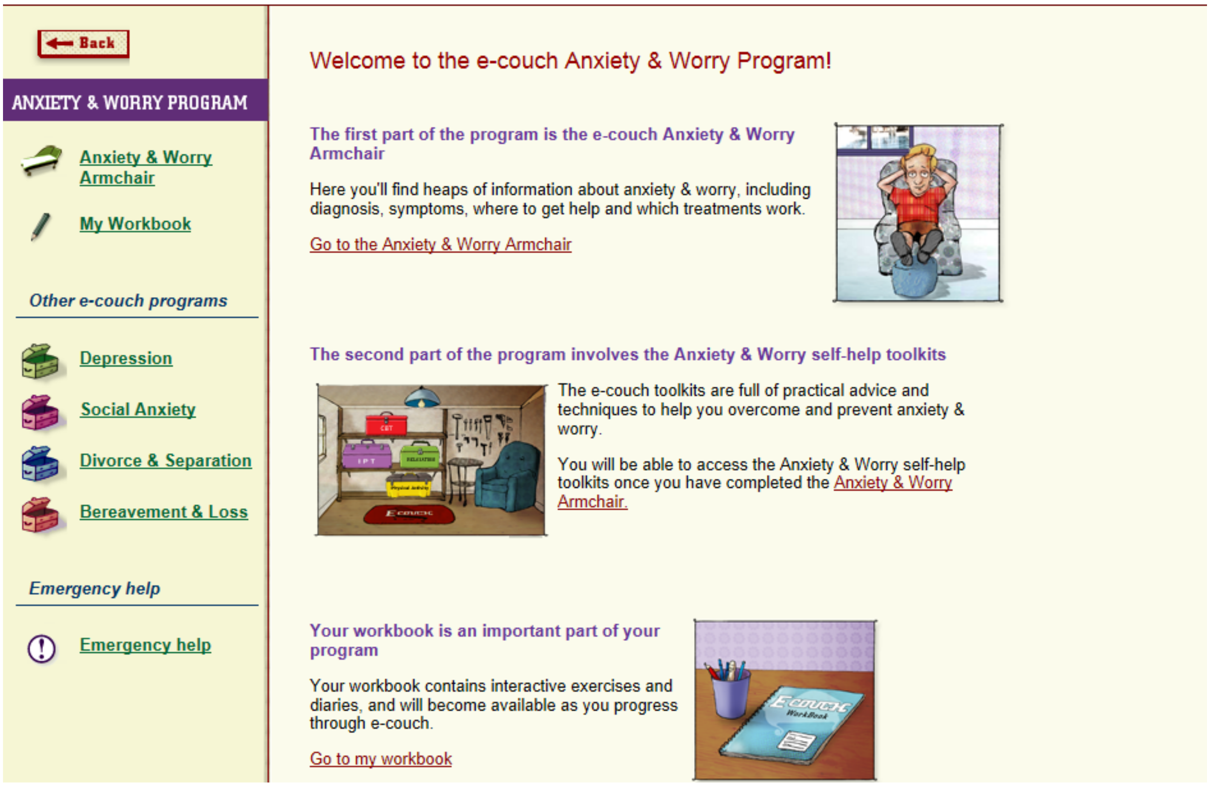

Learn to Live (#10)

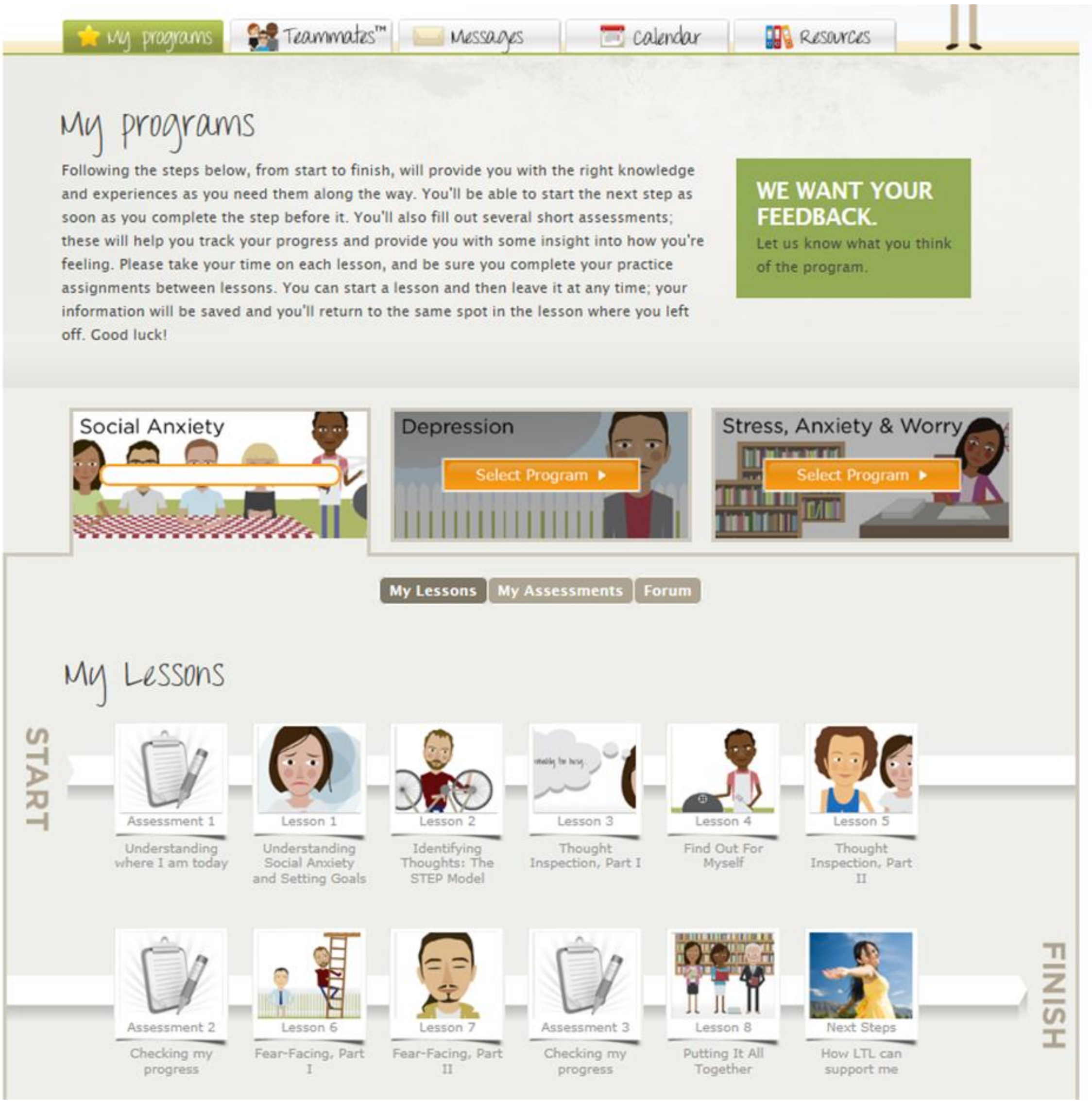

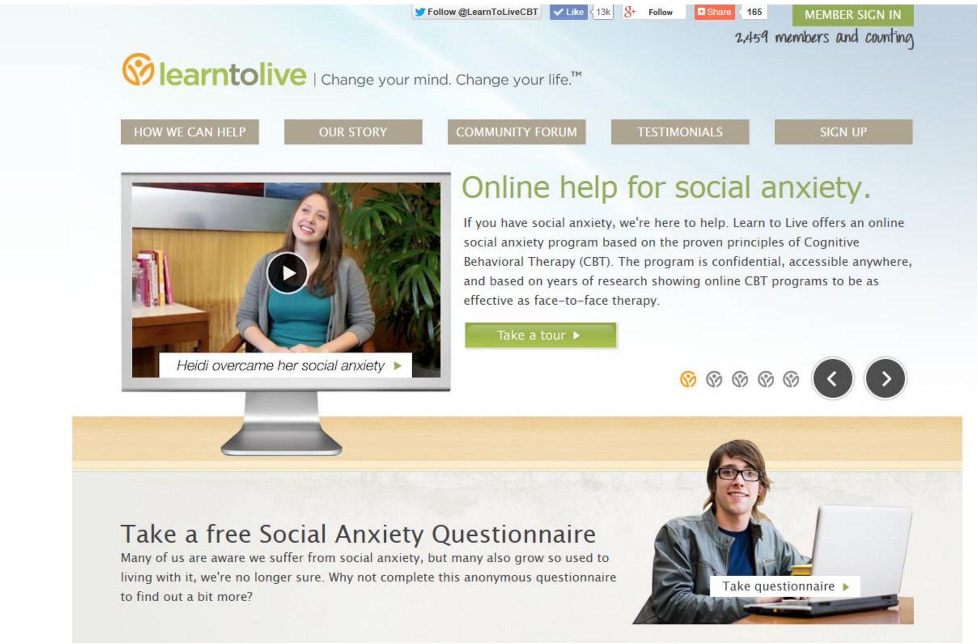

Livanda - Free from Anxiety (#11)

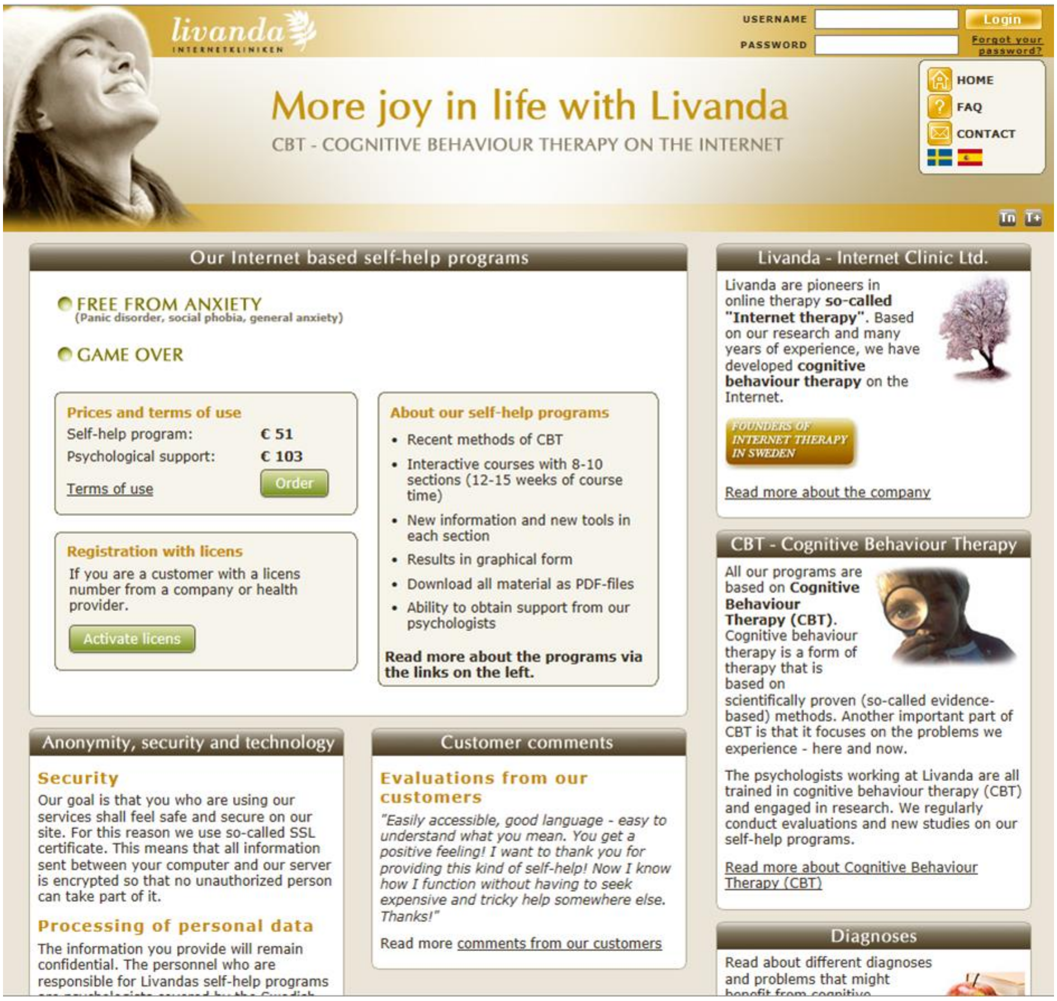

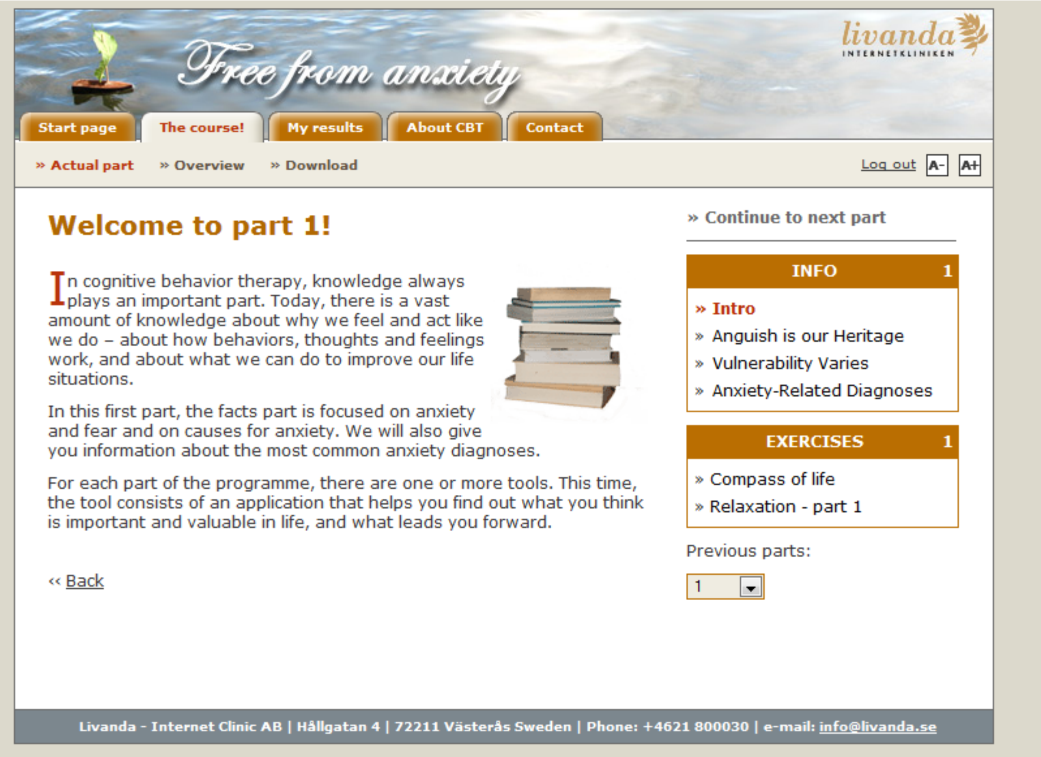

Living Life to the Full (#12)

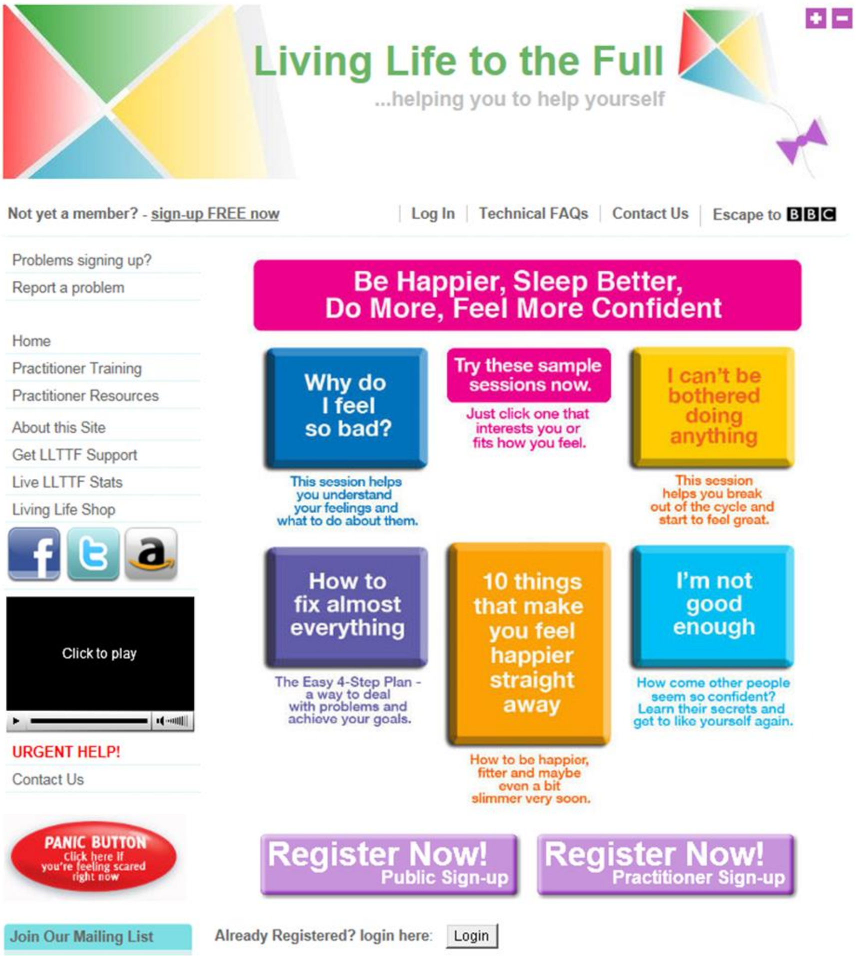

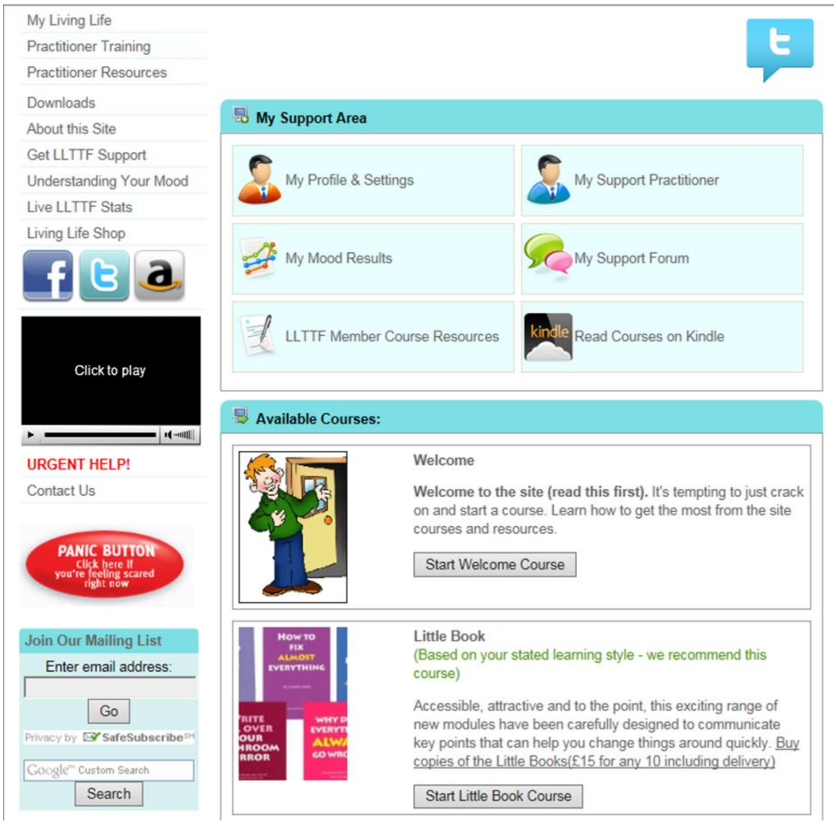

Mental Health Online - Generalised Anxiety Disorder (#13, #14, #15)

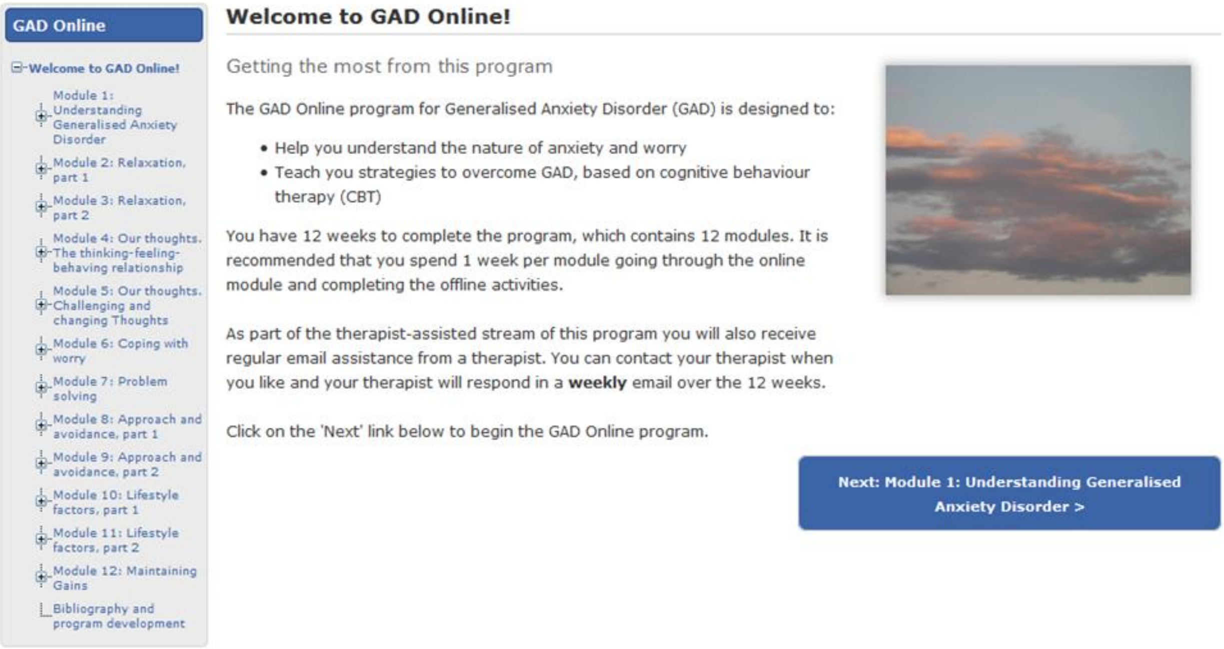

Mood Control (#16)

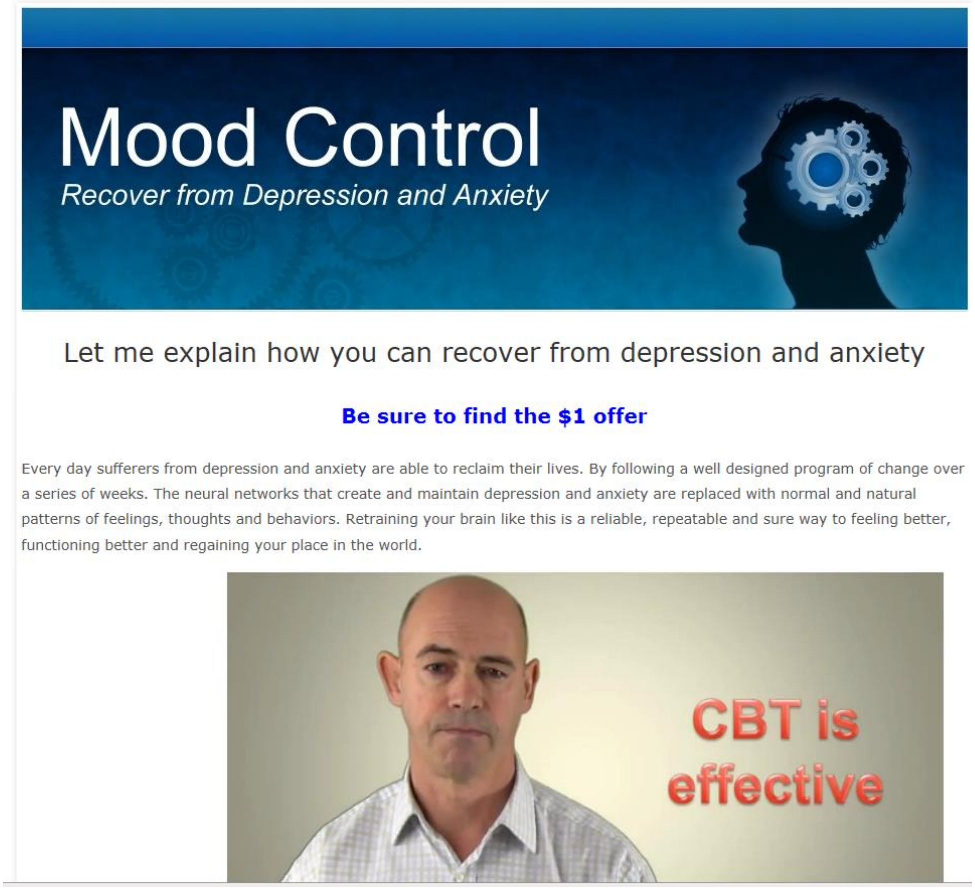
Mood Control
Recover from Depression and Anxiety
Let me explain how you can recover from depression and anxiety
Be sure to find the $1 offer
Every day sufferers from depression and anxiety are able to reclaim their lives. By following a well designed program of change over a series of weeks. The neural networks that create and maintain depression and anxiety are replaced with normal and natural patterns of feelings, thoughts and behaviors. Retraining your brain like this is a reliable, repeatable and sure way to feeling better, functioning better and regaining your place in the world.
CBT is effective

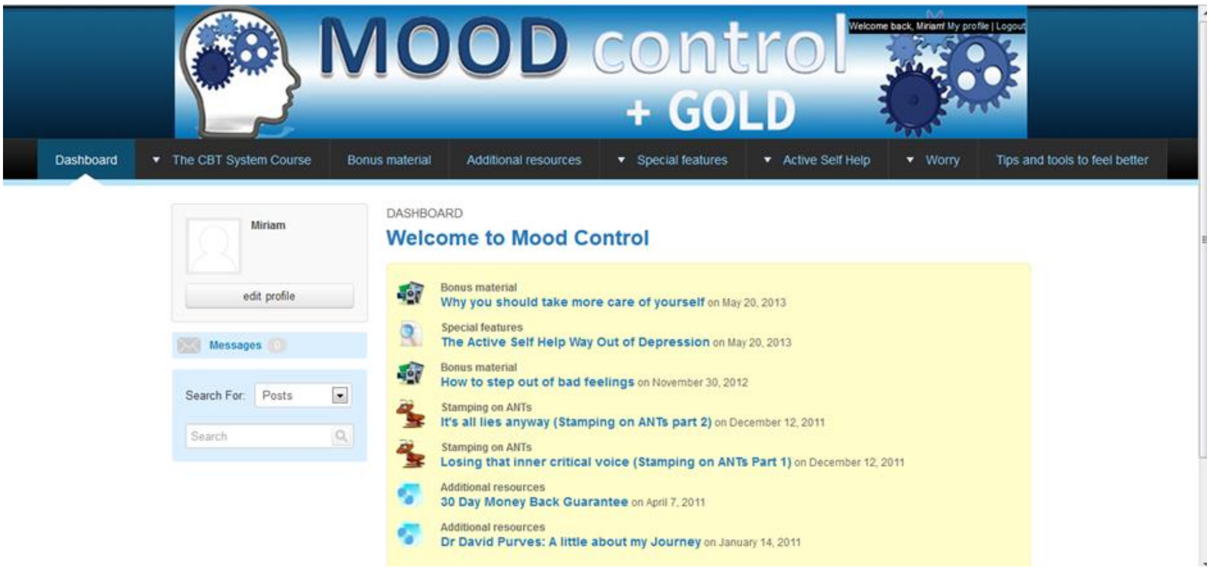
MOOD control + GOLD
Welcome back, Miriam! My profile | Logout
Dashboard
The CBT System Course
Bonus material
Additional resources
Special features
Active Self Help
Worry
Tips and tools to feel better
Miriam
edit profile
Messages
Search For: Posts
Search
DASHBOARD
Welcome to Mood Control
Bonus material
Why you should take more care of yourself on May 20, 2013
Special features
The Active Self Help Way Out of Depression on May 20, 2013
Bonus material
How to step out of bad feelings on November 30, 2012
Stamping on ANTs
It's all lies anyway (Stamping on ANTs part 2) on December 12, 2011
Stamping on ANTs
Losing that inner critical voice (Stamping on ANTs Part 1) on December 12, 2011
Additional resources
30 Day Money Back Guarantee on April 7, 2011
Additional resources
Dr David Purves: A little about my Journey on January 14, 2011

MoodGym (#17)

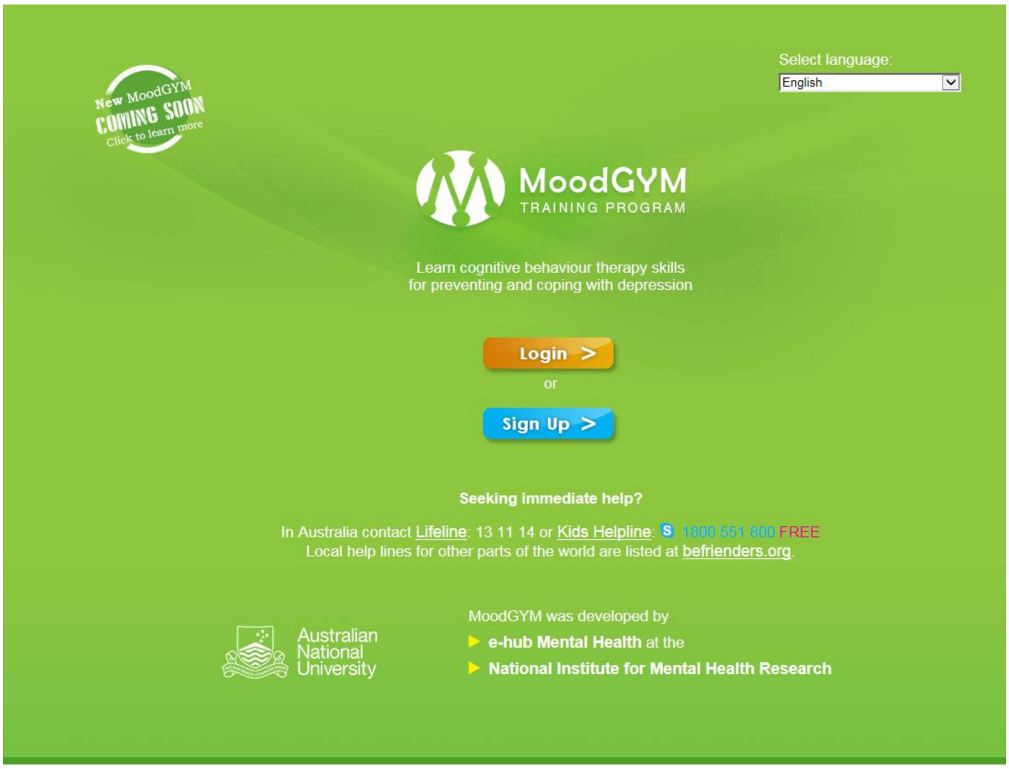

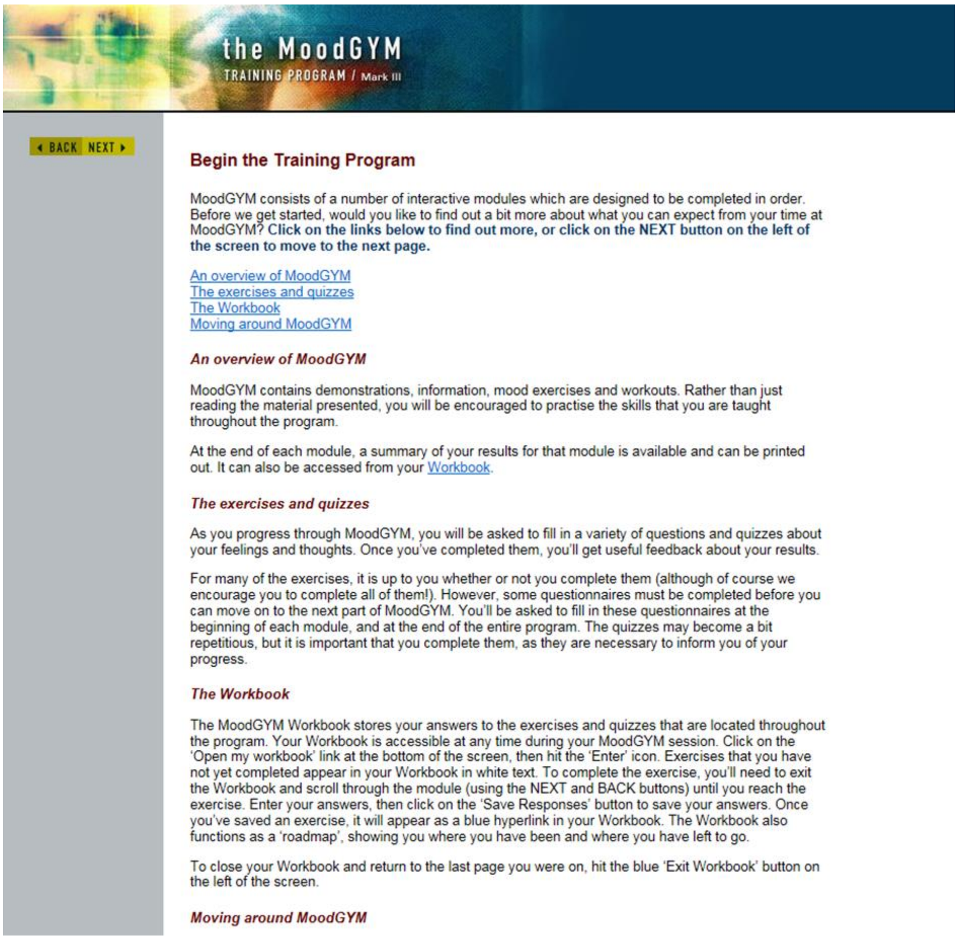

myCompass (#18)

myCompass

Home Help

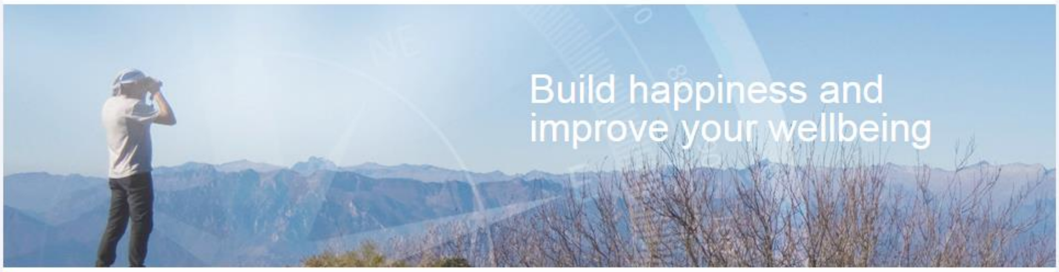

Did you know...

Some people find it hard to achieve the goals they set for the New Year. This can lead to disappointment and frustration and make it hard to stay motivated. Research suggests that having a clear picture or image of your goal in mind can help.

Try our Setting SMART Goals module for more suggestions on how to reach your goals.

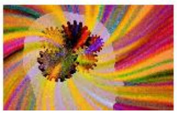

What is myCompass?

Nearly half of all Australians aged 16 and over will suffer from some form of stress, anxiety or depression in their lifetime and the numbers are growing.

myCompass is an interactive self-help service that aims to promote resilience and wellbeing for all Australians. myCompass is a guide to good mental health – it points you in the right direction. You can track your moods, write about them and view information and tips. You can also choose to do one of the modules designed to help you manage mild to moderate stress, anxiety and depression.

To get the most out of myCompass we recommend you:

- track at least 2 moods, feelings or events each day
- complete at least 2 of the modules & home tasks
- use the program regularly for 6 to 8 weeks

Who can use myCompass?

Anyone aged 18years and over can use myCompass. The mobile phone functions can only be used by Australian residents.

Watch Video Overview

Log In

Email or Mobile Number

Password

Forgot Password?

Submit

New Here?

It only takes a moment to register, then you can start using myCompass.

Register

Managing Fear & Anxiety

Anxiety is 'fear that lasts' – and too much anxiety interferes with our well-being and enjoyment of life. First this module helps you analyse what sets off your anxiety and what maintains it. Understanding your anxiety is the first key to managing it. Then it explores the steps you can take to prevent or manage anxiety so that it doesn't take over.

Who is it for?

This module is for people who would like to improve the way they manage anxiety in their lives. We know that some anxiety is normal and useful if it motivates us to action. But sometimes we find ourselves feeling anxious in situations where we don't need to be; it's as if the body doesn't re-set itself to a calm state once the threat or danger is past. We call this having mild to moderate anxiety difficulties.

Why should you do it?

While anxiety isn't harmful, it feels very unpleasant, it can interfere in our lives and when extreme it doesn't really help us solve problems. People can develop anxiety for a number of reasons, usually related to their life circumstances and current stresses. Some people are just more prone to developing anxiety; it 'runs in the family'. Regardless of how your anxiety developed, you can learn to understand and manage it better. This module encourages you to develop an awareness of what sets off your anxiety, so that you can take steps to prevent or prepare for it.

Module Structure

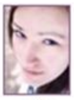
Kaz

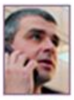
Costa

Duration: 2-3 weeks

Home tasks: Leave a week between the module sessions to complete the home tasks and apply the module information to your situation. If you don't do the home tasks you may miss out on finding out something new about yourself.

Session 1	Session 2	Session 3
**Areas Covered:** Introduction to Managing Fear & Anxiety; Anxiety warning signs; Anxiety disorders; How to recognise anxiety; Understanding the situational triggers of anxiety. 16 minutes	**Areas Covered:** The places, people and things that affect your anxiety; Managing anxiety through breathing; Understanding the thoughts and other factors that lead to anxiety. 16 minutes	**Areas Covered:** The thoughts and other factors that trigger anxiety; Identifying unhelpful thoughts. 10 minutes
**Home task:** Monitoring of 3 anxiety episodes. 6 minutes per episode.	**Home task:** Monitoring another 3 anxiety episodes. 6 minutes per episode	

Modules List

Managing Fear & Anxiety
Setting SMART Goals
Increasing Pleasurable Activities
Sleeping Well
Managing Loss & Major Life Changes
Breathing & Relaxation
Managing Stress & Overload
Communicating Clearly
Solving Problems
Building Happiness & Wellbeing
Tackling Unhelpful Thinking
Taking Charge of your Worry

Further Reading

Take control of your worry, Dr Lisa Lampe

Online Therapy (#19, #20, #21 , #22 , #23 , #24)

Online-Therapy.com

Are you a therapist? | Client log in

OUR PROGRAMS | HOW IT WORKS | FAQ | REVIEWS | ABOUT

Overcome Your Anxiety with Online Therapy

Now only! £19.95 /week

Anxiety » Test » How it works » Reviews » Get Started »

There are so many advantages when getting help using online therapy compared to traditional face-to-face therapy. It is more affordable, accessible, anonymous and time saving to just name a few. Right now you pay as little as **£19.95 per week** for full access to:

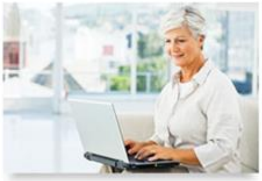

- ✓ Your own qualified therapist; communicate daily via worksheets + access to chat
- ✓ Evidence-based online therapy program
- ✓ Easy-to-follow information & tools in each section
- ✓ Forum to get help from others
- ✓ Tests to see your progress

To get the best out of the program we recommend that you use our services during 8 weeks, but that is totally up to you. You decide if you need help for a longer or shorter time. Our therapy for anxiety is based on cognitive behavioral therapy (CBT).

Online CBT for Anxiety
It's not external events that trigger your anxiety; instead it's how you feel and perceive the events that cause your anxiety. Using our online therapy for anxiety you'll learn how to identify your negative thinking patterns that cause your anxiety and replace them with realistic and more positive thoughts. CBT online will help you learn to recognize when you're anxious and how this feels emotionally and physically, but more importantly your will learn coping skills to neutralize this anxiety. Your therapist will help you to gradually and repeatedly expose yourself to the situations and fears that normally trigger your anxiety. Eventually you will be able to go through these events without experiencing the anxiety that up to this point has limited your quality of life.

Click on the links below to learn more or to get started right away.

How it works > | Get Started! >

MY THERAPY | LOG OFF

Online-Therapy.com

Worksheet: Session 3 - My unhealthy behaviors

Unhealthy behaviors
Our unhealthy behaviors work as coping strategies and whilst they may seem effective at first, they are actually just causing damage over the longer term by fueling the anxiety. Write down your unhealthy behaviors.

You wrote: 2014-04-29 (15:50)

Only sleeping a few hours per night
Not eat enough food
Overload myself with work

Add a new comment:

< Back (don't save) | Continue >

Serenity Program - Anxiety Program (#25)

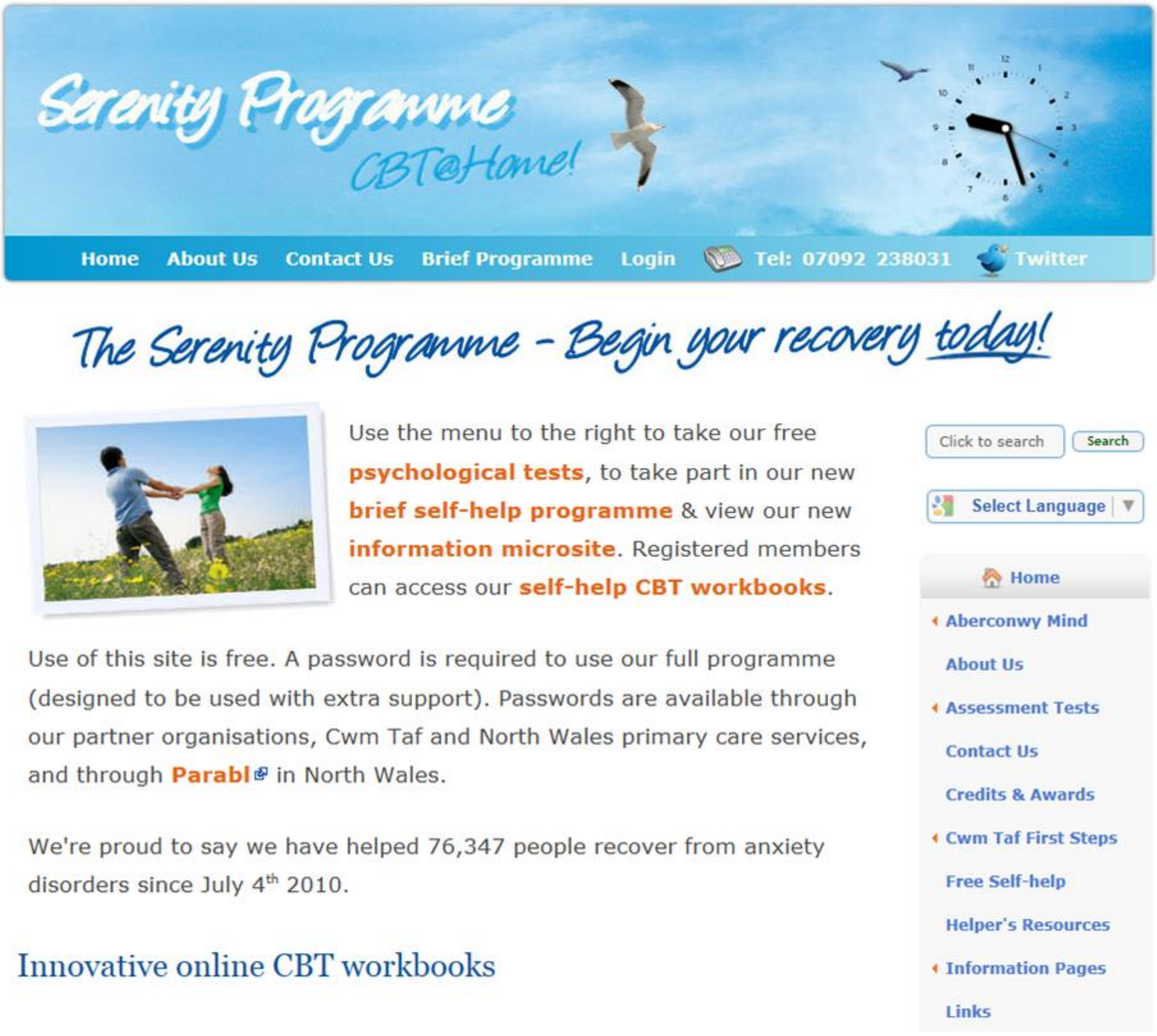

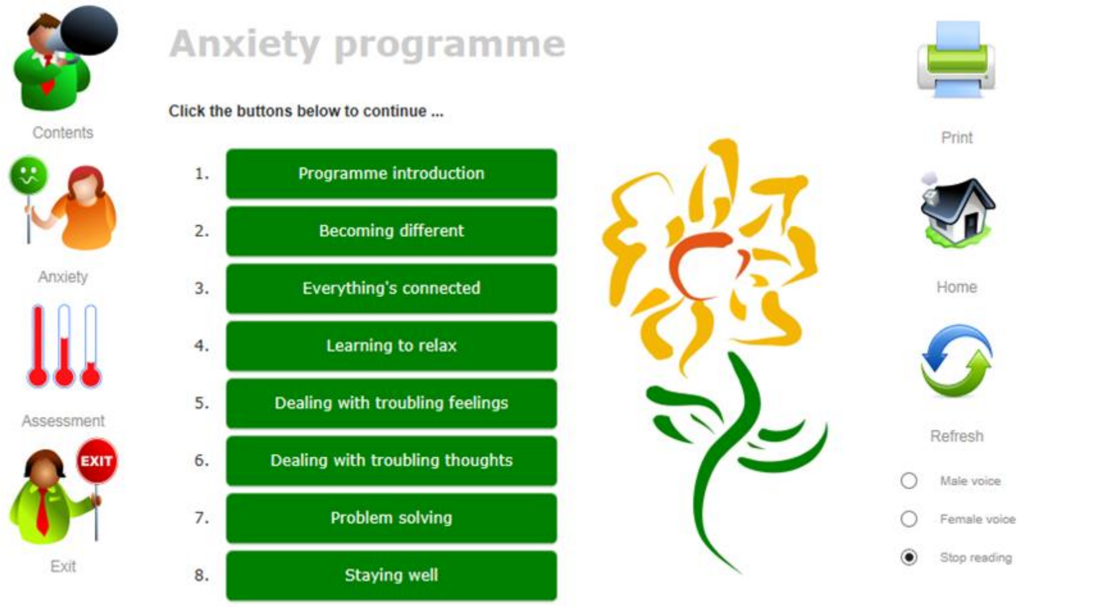

Social Anxiety Institute (#26)

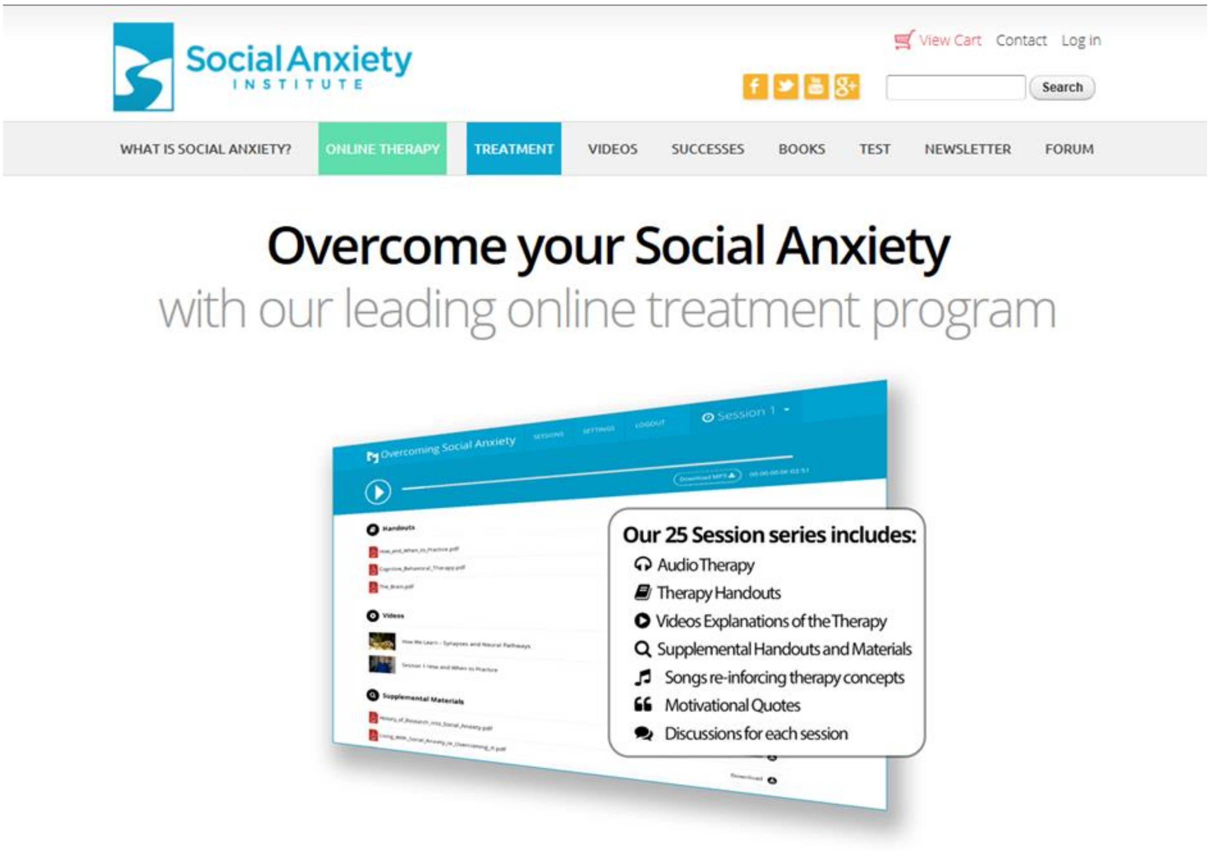

This Way Up Clinic (#27, #28 , #29, #30)
This Way Up Self-help
(#31, #32)
This Way Up School (#33, #34)

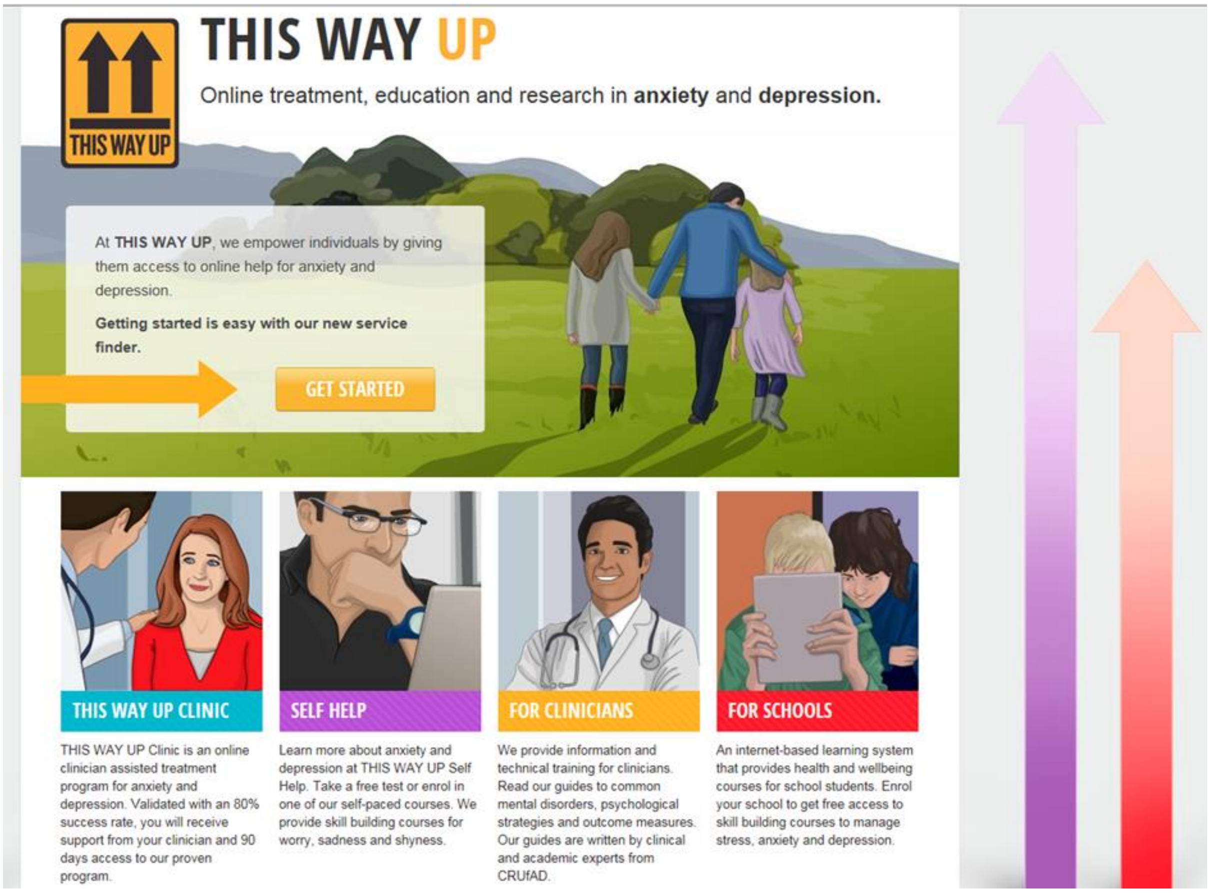

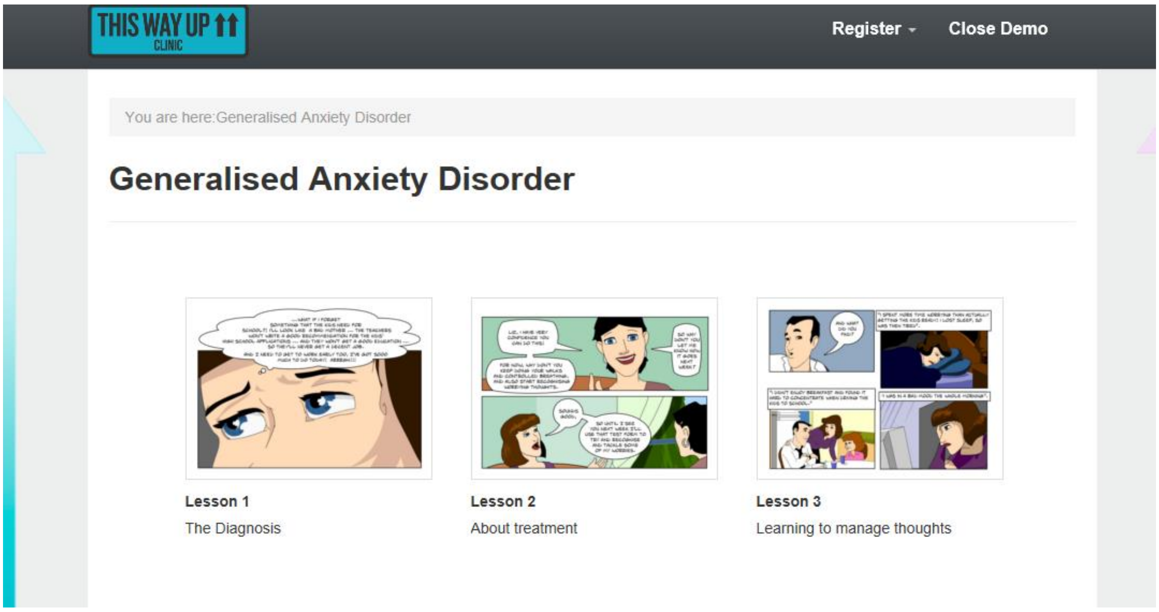

Supplement: Multimedia Appendix 2 [file mental_v3i2e14_app2.pdf]
